# Smarcad1 mediates microbiota-induced inflammation in mouse and coordinates gene expression in the intestinal epithelium

**DOI:** 10.1186/s13059-020-01976-7

**Published:** 2020-03-11

**Authors:** Juri Kazakevych, Jérémy Denizot, Anke Liebert, Mariana Portovedo, Mia Mosavie, Payal Jain, Claudia Stellato, Claire Fraser, Renan Oliveira Corrêa, Marina Célestine, Raphaël Mattiuz, Hanneke Okkenhaug, J. Ross Miller, Marco Aurélio Ramirez Vinolo, Marc Veldhoen, Patrick Varga-Weisz

**Affiliations:** 1 Nuclear Dynamics, Babraham Institute, Cambridge, CB22 3AT UK; 2 Present Address: Université Clermont Auvergne, Inserm U1071, INRA USC2018, M2iSH, F-63000 Clermont–Ferrand, France; 3 Present Address: The Francis Crick Institute, London, NW1 1AT UK; 4 Laboratory of Immunoinflammation, Institute of Biology, UNICAMP, Campinas, 13083-862 Brazil; 5 School of Biological Sciences, University of Essex, Colchester, CO4 3SQ UK; 6 Imaging Facility, Babraham Institute, Cambridge, CB22 3AT UK; 7 Lymphocyte Signalling and Development, Babraham Institute, Cambridge, CB22 3AT UK; 8 Present Address: Instituto de Medicina Molecular | Joâo Lobo Antunes, Faculdade de Medicina da Universidade de Lisboa, 1649-028 Lisbon, Portugal

## Abstract

**Background:**

How intestinal epithelial cells interact with the microbiota and how this is regulated at the gene expression level are critical questions. Smarcad1 is a conserved chromatin remodeling factor with a poorly understood tissue function. As this factor is highly expressed in the stem and proliferative zones of the intestinal epithelium, we explore its role in this tissue.

**Results:**

Specific deletion of *Smarcad1* in the mouse intestinal epithelium leads to colitis resistance and substantial changes in gene expression, including a striking increase of expression of several genes linked to innate immunity. Absence of Smarcad1 leads to changes in chromatin accessibility and significant changes in histone H3K9me3 over many sites, including genes that are differentially regulated upon *Smarcad1* deletion. We identify candidate members of the gut microbiome that elicit a Smarcad1-dependent colitis response, including members of the poorly understood TM7 phylum.

**Conclusions:**

Our study sheds light onto the role of the chromatin remodeling machinery in intestinal epithelial cells in the colitis response and shows how a highly conserved chromatin remodeling factor has a distinct role in anti-microbial defense. This work highlights the importance of the intestinal epithelium in the colitis response and the potential of microbial species as pharmacological and probiotic targets in the context of inflammatory diseases.

## Background

The intestinal epithelium is a highly dynamic tissue that is constantly renewed within a few days, driven by intestinal stem cells that reside at the base of intestinal crypts [1, 2]. Intestinal stem cells and their derivatives express many factors involved in DNA replication, DNA repair, chromatin packaging, and chromatin remodeling, including ATP-dependent remodeling factors [3]. The roles of these factors in genomic and epigenomic stability of this tissue are likely of great importance. Understanding epithelial biology is complicated by the fact that this tissue is in close proximity to the gut microbiota, which normally is either innocuous or even beneficial to the host, but can become pathogenic. How intestinal epithelial cells interact with the host immune system and the microbiota and how this is regulated at the gene expression level are critical questions.

ATP-dependent chromatin remodeling factors are key regulators of many genome functions, including transcription, DNA repair, and replication [4, 5]. The Smarcad1/Etl1/Fun30 family of chromatin remodeling factors is one of the most highly conserved families, found from fission (Fft1-3) and budding yeast (Fun30) to mouse (Smarcad1) and human (SMARCAD1), and is defined by the presence of CUE-domains in addition to the helicase-like domains [5]. Smarcad1 and its homologs have roles in gene regulation including gene silencing [5–9], heterochromatin maintenance [10, 11], genome organization [12, 13], and DNA repair [14, 15]. A mutation in a skin-specific isoform of SMARCAD1 in humans causes adermatoglyphia (the loss of finger prints) and Basan syndrome, which affects skin integrity [16–18]. *SMARCAD1* haploinsufficiency has also been linked to another skin abnormality, the Huriez syndrome [19].

While an early study showed that Smarcad1 is not required for mouse ES cell viability or proliferation [20], several studies linked Smarcad1 to stem cell biology [21–23]. A full non-conditional knockout (KO) of Smarcad1 using an exon-trap strategy indicated that while Smarcad1 was not essential for development, its absence caused impaired postnatal viability, reduced fertility, and skeletal dysplasia [20]. In order to explore the role of Smarcad1 in the mouse further, we generated a new conditional deletion model and focused on the role of this gene in the intestinal epithelium. Our analysis shows that Smarcad1 deletion affects histone modifications in this tissue, modifying gene expression and intestinal epithelium-microbiome interactions, which, in turn, impinges on the colitis response in a DSS-induced mouse model.

## Results

### A novel Smarcad1 deletion model

**Fig. 1:**
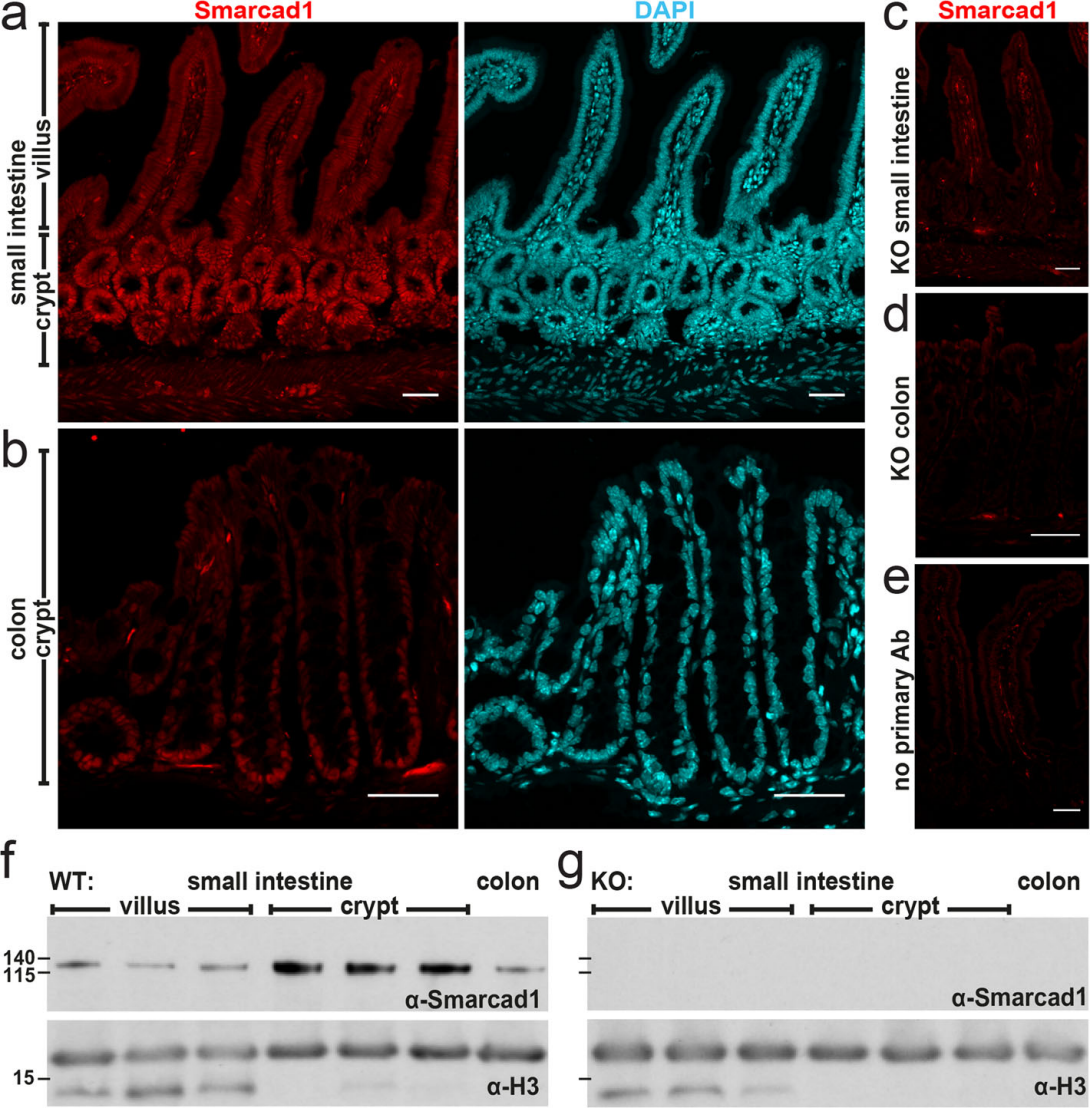
Smarcad1 localization in the intestinal tract and *Vil-cre*-mediated KO. **a**–**e** Intestinal epithelium localization of Smarcad1 (red) by IF staining and nuclear counterstaining with DAPI (cyan) shown as maximum intensity projections. Scale bars, 40 μm. **a** Wild type (WT) small intestinal (SI) epithelium. **b** WT colon epithelium. **c**, **d***Vil-cre*-mediated tissue-specific knockout of *Smarcad1* (KO) in SI (**c**) and colon (**d**). **e** No primary antibody control on WT SI. **f**, **g** Representative Western blots of Smarcad1 protein levels in the intestinal tract and H3 loading normalization in WT (**f**) and KO (**g**) samples. Villus and crypt SI samples are shown as anterior, central, and posterior fractions of the SI. Protein size markers [kDa] indicated on the left. Quantitation of Western blots (*n* = 3) is shown in Additional file 1: Fig. S1b

We generated a conditional Smarcad1 deletion model via recombineering to include *loxP* sites into the mouse Smarcad1 gene in C57BL/6 J-derived ES cells, using a cre-recombinase for excision in C57BL/6 J-strain background animals. We framed exons 12–14 with *loxP* sites as these exons code for amino acids critical for ATP binding of Smarcad1, and thus, their deletion should abrogate enzymatic activity. Furthermore, deletion of these exons causes a frame shift mutation, leading to the expression of no functional protein beyond exon 14. In fact, we found that deletion of these exons led to no detectable protein in various cell types (Fig. 1). As there is evidence of a short transcript of SMARCAD1 in humans, transcribed from an internal start site compared to the full-length transcript [16], we reasoned that our deletion strategy would insure complete deletion of any functional Smarcad1.

### Smarcad1 is highly expressed in the intestinal crypt, and its deletion affects epithelial gene expression

RNA-seq, immunohistochemistry, and Western blot analysis show that *Smarcad1* is highly expressed in the proliferative zones of the intestinal epithelium, both in the small intestine and colon (Fig. 1a, b, f, Additional file 1: Fig. S1a, b, Additional file 2: Table S1 for statistical data). In order to explore a role of Smarcad1 in this tissue, we monitored the effects of *Villin-cre*- (*Vil-cre*) mediated [24] tissue-specific Smarcad1 abrogation (*Villin-Cre Smarcad*^*fl/fl*^, further referred as *Smarcad1*-KO) (Fig. 1, Additional file 1: Fig. S1c). Using EdU pulse labeling, we did not find evidence of a role of Smarcad1 in regulating dynamics of cell proliferation in this tissue (Additional file 1: Fig. S1d-f). We did not detect changes in the barrier function by the FITC-dextran assay (Additional file 1: Fig. S1g).

**Fig. 2:**
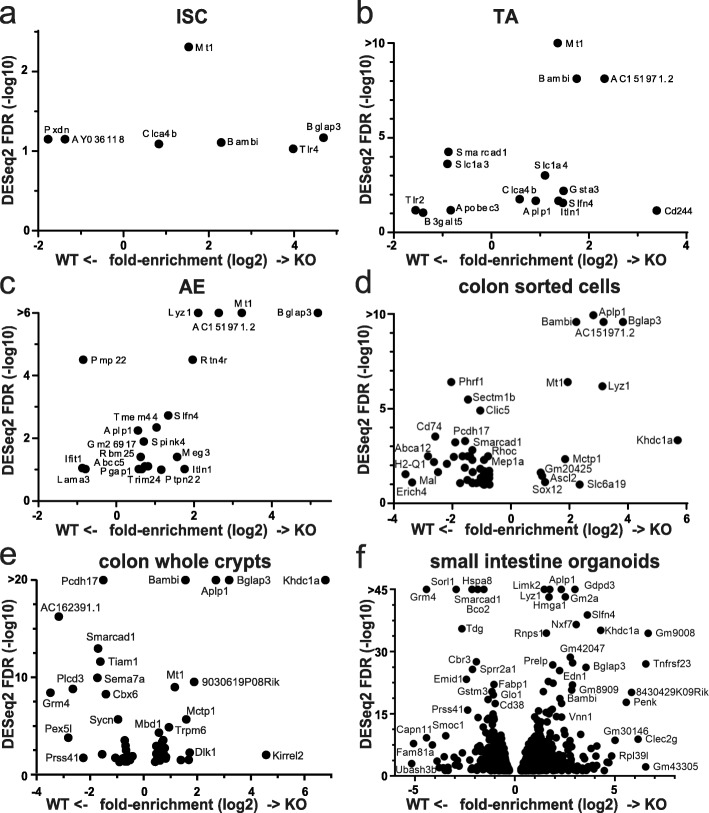
Gene expression changes on *Smarcad1*-KO. Significantly up/downregulated genes (DESeq2 test with cutoff FDR < 0.1 for **a**–**d** and FDR < 0.05 for **e**, **f**; for additional data and annotations, see Additional file 3: Table S2, Additional file 4: Table S3) in RNA-seq experiments (*n* = 3). *X*-axis: *Smarcad1*-KO/WT log (2) fold-enrichment of reads per transcript. *Y*-axis: -log (10) FDR. High FDR values capped at indicated maximum *Y* values for visualization. 0-expression values were set to 0.1 to allow log-plotting of fold changes. **a** Intestinal stem cells (ISC), FACS-selected by high *Lgr5*-GFP signal. **b** Transit amplifying cells (TA), FACS-selected by low *Lgr5*-GFP signal. **c** Adult enterocytes (AE), FACS-selected by EPCAM-positive and CD31/45-negative signal. **d** Colon epithelium, FACS-selected by EPCAM-positive and CD31/45-negative signal. **e** Colon epithelium, isolated as whole crypt suspension. **f** Small intestine-derived organoid culture

We performed gene expression profiling by mRNA-seq from whole small intestinal tissue, extracted colon crypts, and sorted stem, proliferative, and adult enterocytes from the small intestine (Fig. 2a–e, Additional file 1: Fig. S1a, Fig. S4e, Additional files 3: Table S2, Additional file 4: Table S3, Additional file 5: Table S4). We also performed mRNA-seq on small intestinal organoids isolated from mice where *Smarcad1* had been deleted and controls, to assess microbiota- and immune system-independent gene expression (Fig. 2f). The transcriptome analysis in organoids identified the largest number of differentially expressed genes (DEG, 1407, *p* < 0.05, Fig. 2f, Additional file 3: Table S2). In these datasets, we noted differential expression, mostly upregulation, of several genes linked to innate immunity and the epithelium interaction with microbiota in KO (Additional file 1: Fig. S2). These genes include *Tlr4*, encoding a Toll-like receptor (intestinal stem cells and organoid datasets); *Itln1*, a lectin receptor; defensins *Defa22* and *Defa26*; *Wdfy1* (positively regulates TLR3/4 signaling pathways [25]); and anti-microbial protein genes *Ang4* [26], *Reg3b*, and *Lyz1* (lysozyme). *Mt1* that is significantly upregulated on *Smarcad1* deletion plays an important role in the prevention of colonic mucosal inflammation in the dextran sodium sulfate (DSS)-induced mouse model of colitis [27]. Interestingly, *Mt1* is not significantly upregulated in the small intestinal organoid culture upon *Smarcad1*-KO, indicating that this upregulation may depend on some external cue, such as niche, microbiota, or immune cells (Fig. 2, Additional file 1: Fig. S2).

Remarkably, one gene, *Bglap3* (also called *Bglap-rs1*), whose expression was most enhanced in the small intestine and colon crypt datasets compared to control, was upregulated in all datasets upon *Smarcad1*-KO (Additional file 1: Fig. S3a, b). Consistent with increased expression of *Bglap3*, we found increased protein levels of osteocalcin by Western blot (Additional file 1: Fig. S3c-e). This gene codes for an osteocalcin protein of a class that is normally predominantly expressed in bone by osteoblasts, regulating calcification, and can also be secreted and acts like a hormone, coordinating bone metabolism with body physiology [28]. The role of *Bglap3* in the gut (if any) is not clear, but one might speculate that this protein may regulate intestinal calcium uptake. Calcium particles are known to be generated and secreted in the gut and have been linked to gut immunity by aiding the delivery of antigens to Peyer’s patches [29]. We tested this hypothesis by assessing Ca^2+^ blood levels, showing that they do not change on upregulation of osteocalcin in the small intestine upon *Smarcad1*-KO, suggesting a separate function of osteocalcin in this tissue (Additional file 1: Fig. S3f).

By comparing the various transcriptome analyses (from colon (CO), sorted stem (ISC), and transit amplifying (TA) cells as well as adult enterocytes (AE) from small intestine and small intestine organoids (ORG)), we derived a list of genes that are upregulated repeatedly in these analyses and, thus, represents a gene expression hallmark reflecting *Smarcad1* deletion (Fig. 2, Additional file 1: Fig. S2, Additional file 3: Table S2, Additional file 4: Table S3). In addition to *Bglap3* (significant DEG in ISC, AE, ORG, CO), *Mt1* (ISC, TA, AE, CO), and *Lyz1* (AE, ORG, CO), these genes include *Bambi* (ISC, TA, ORG, CO), *Aplp1* (TA, AE, ORG, CO), *Slfn4*, *Itln1* (TA, AE, ORG), *Kirrel2*, *Tiam1*, *Khdc1a*, and *Mbd1* (ORG, CO). *Bambi* (BMP and activin membrane-bound inhibitor) is generally thought to function as an inhibitory pseudo- (decoy) receptor for TGFβ/BMP signaling pathways, and thus, its overexpression may affect inflammatory responses [30–34]. *Slfn4* is expressed from a cluster of genes all expressing Schlafen family members. These are AAA-domain containing proteins with various roles, including in regulating cell proliferation, in immune system development, function, and interferon response, and recently, these proteins have been suggested to be involved in RNA metabolism [35] (reviewed in [36, 37]). *Aplp1* codes for an amyloid precursor-like protein and is normally primarily expressed in the nervous system [38]. *Kirrel2* codes for a glycoprotein that regulates insulin secretion in beta cells in the pancreas [39]. *Tiam1* (T lymphoma invasion and metastasis 1) codes for a guanine nucleotide exchange factor (GEF) of Rac1 involved in many signaling pathways (reviewed in [40]). *Khdc1a* codes for a translational repressor involved in endoplasmic reticulum-dependent apoptosis [41]. In summary, the transcriptomic analysis indicates that Smarcad1 is involved in repression of genes linked to innate immunity and inflammation. Furthermore, Smarcad1 appears to control the expression of genes such as *Aplp1* and *Bglap3* that are not normally associated with intestinal function.

### Smarcad1 impacts H3K9me3 over genes and regulatory elements and controls regulatory element accessibility

**Fig. 3:**
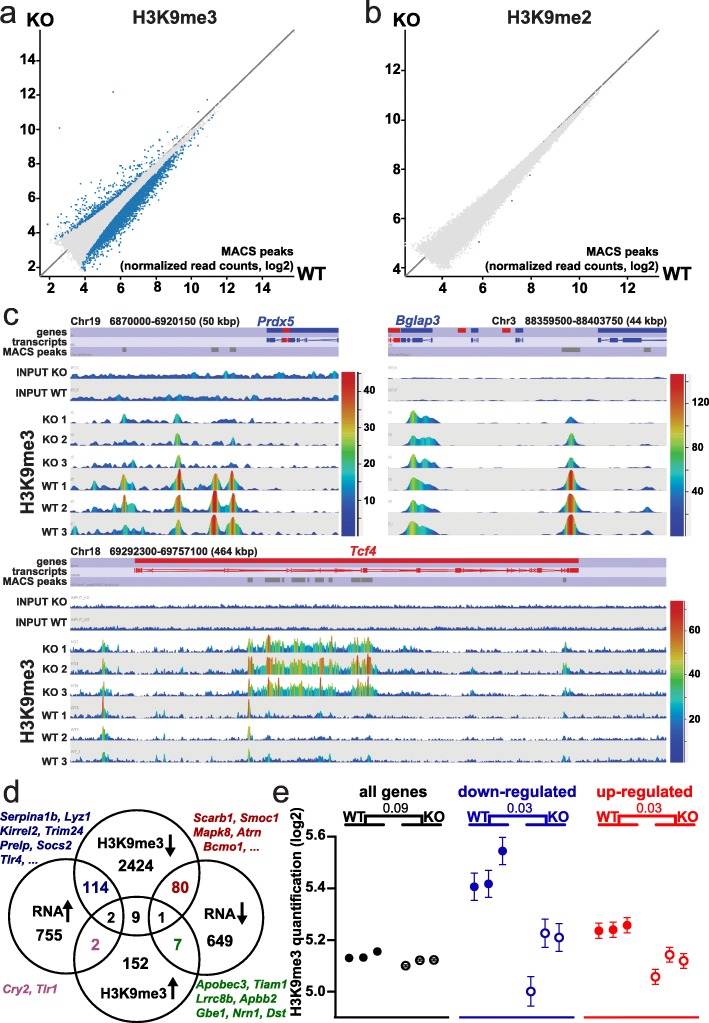
H3K9me2/3 changes linked to *Smarcad1*-KO in the small intestine. EdgeR analysis (*p* < 0.05) of read counts over H3K9me3 (**a**) or H3K9me2 (**b**) peaks reveals regions with significant alterations in these histone marks on deletion of Smarcad1 in the small intestinal epithelium by *Vil-cre* (KO). Datasets shown as averaged from 3 replicates (for additional data and annotations, see Additional file 6: Tables S5, Additional file 7: Table S6). **c** Examples of H3K9me3 changes over selected regions. The top annotation tracks indicate genes, transcripts, and H3K9me3 MACS peaks. Read counts are shown in linear scale, color coded. Chromosomal positions and window sizes in kbp are indicated. **d** Link between changes in gene expression and changes in H3K9me3. Venn diagrams showing the distribution of differentially expressed genes (DEG) in small intestine organoids on *Smarcad1*-KO (see Additional file 1: Fig. S2a, Additional file 3: Table S2) and genes from small intestinal crypts with differential H3K9me3 MACS peaks within ± 5 kbp of the gene (EdgeR *p* < 0.05, see Additional file 6: Table S5). There is a significant overlap between genes with increased expression and decreased H3K9me3 (*p* = 0.00017, chi-square, number of expressed genes 25,965, Additional file 2: Table S1). The Venn diagrams are not drawn to scale. **e** Normalized H3K9me3 ChIP-seq read count quantitation over MACS peaks, log2 transformed, adjusted for matching distributions in SeqMonk. Separate quantitations over all annotated genes (32,029 genes) and ± 5 kbp up- and downstream of genes with up- and downregulated expression in small intestinal organoids on *Smarcad1*-KO (1420 genes, see Additional file 1, Fig. S2, Additional file 3: Table S2). *p* values from an unpaired two-tailed *t* test with Welch’s correction are indicated (*n* = 3). Error bars indicate the standard error of the mean (SEM) of read count quantitation of each biological replicate

Previously, we have shown that Smarcad1 promotes heterochromatin features globally in proliferating cells in culture [10], including histone modifications H3K9me3 and H3K9me2 as well as HP1 (Heterochromatin Protein 1) chromatin binding. Depletion of Smarcad1 conversely promoted global histone acetylation, including H3K9ac [10]. However, when we examined H3K9me2 and H3K9me3 levels in tissue extracts from crypts and villi of the small intestine, as well as colon epithelium extracts, we did not find global changes of H3K9me2/3 upon deletion of Smarcad1, except for some drop in H3K9me2 in the small intestinal crypts (Additional file 1: Fig. S4a-c). In order to test if Smarcad1 has a role in repressive chromatin on a more local level, we performed ChIP-seq for H3K9me2 and H3K9me3 on chromatin extracts of small intestinal crypts. Consistent with the notion that H3K9me3 is linked to gene repression, we found that this mark is rather low over promoters of highly expressed genes compared to promoters of lowly expressed genes (Additional file 1: Fig. S4d). The majority of sites (identified as MACS-peaks, [42]) with changes of H3K9me3 levels upon *Smarcad1*-KO showed a depletion of this mark with only a minor fraction showing increased levels (Fig. 3a, Additional file 1: Fig. S4f, Additional file 6: Table S5). We found that deletion of *Smarcad1* led to a drop in H3K9me3 peaks close to and within many genes, suggesting their position over regulatory elements such as promoters and enhancers (Fig. 3c, Additional file 1: Fig. S4f). We observed that there is a significant association between changing gene expression upon *Smarcad1*-KO and changes in H3K9me3 (Fig. 3d) and that genes that are misregulated in the small intestine on *Smarcad1*-KO are in general associated with decreased H3K9me3 levels (Fig. 3d, e). Areas that exhibit an increase of H3K9me3 are usually much broader and, importantly, are found within the transcribed region of genes (Fig. 3c, Additional file 1: Fig. S5). Some of the genes that show this type of increase in H3K9me3 in their gene body show a decrease (e.g., *Tlr2*, *Apobec3*, *Tiam1*) or no change (*Tcf4*, *Fat1*) in expression on *Smarcad1*-KO.

In contrast to H3K9me3, we found that deletion of Smarcad1 did not appear to affect H3K9me2 in a significant manner (Fig. 3b, Additional file 7: Table S6).

**Fig. 4:**
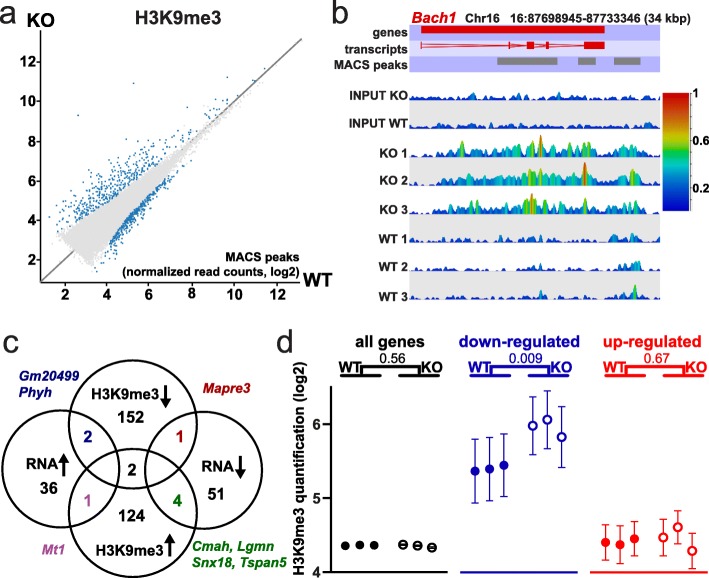
H3K9me3 changes linked to *Smarcad1*-KO in the colon. **a** EdgeR analysis (*p* < 0.05) of read counts over H3K9me3 peaks reveals regions with significant alterations in this histone mark on deletion of *Smarcad1* in the small intestinal epithelium by *Vil-cre* (KO). Datasets shown as averaged from 3 replicates (for additional data and annotations, see Additional file 8: Table S7). **b** Examples of H3K9me3 changes over *Bach1*. The top annotation tracks indicate the gene, exons, and H3K9me3 MACS peaks. Read counts are shown in linear scale, color coded. Chromosomal position and length in kbp are indicated. **c** Link between changes in gene expression and changes in H3K9me3. Venn diagrams showing the distribution of differentially expressed genes (DEG) in the colon on *Smarcad1*-KO (93 DEG in either whole crypt or sorted epithelium datasets, see Additional file 1: Fig. S2b, Additional file 4: Table S3) and genes with differential H3K9me3 MACS peaks within ± 5 kbp of the gene (EdgeR *p* < 0.05, see Additional file 8: Table S7). There is a statistically significant overlap between genes with decreased expression and increased H3K9me3 (*p* = 0.05, chi-square, number of expressed genes 25,965, Additional file 2: Table S1). The Venn diagrams are not drawn to scale. **d** Normalized H3K9me3 ChIP-seq read count quantitation over MACS peaks, log2 transformed, adjusted for matching distributions in SeqMonk. Separate quantitations over all annotated genes (32,029 genes) and ± 5 kbp up- and downstream of genes with up- and downregulated expression in the colon on *Smarcad1*-KO (93 DEG in either whole crypt or sorted epithelium datasets, see Additional file 1: Fig. S2b, Additional file 4: Table S3). *p* values from an unpaired two-tailed *t* test with Welch’s correction are indicated (*n* = 3). Error bars indicate the SEM of read count quantitation of each biological replicate

We additionally performed H3K9me3 analysis on colon epithelium by ChIP-seq, as the colon epithelium functionally differs from the small intestinal epithelium. Similar to the findings in the small intestine, we found strong and numerous changes in H3K9me3 on *Smarcad1-*KO, albeit we detected fewer sites with decrease in H3K9me3 (Fig. 4a–c, Additional file 8: Table S7). Changes were again linked to changes in gene expression on *Smarcad1-*KO, but only significantly over downregulated genes that showed an increase in H3K9me3 (Fig. 4c). In contrast to the small intestine, we did not observe globally a decrease of H3K9me3 over differentially expressed genes and that this mark generally increased over downregulated genes (Fig. 4d). The observed difference between H3K9me3 over upregulated genes in the small intestine versus colon epithelium may relate to the fact that proportionally, we observe more sites where H3K9me3 decreases in the small intestine compared to colon epithelium.

**Fig. 5:**
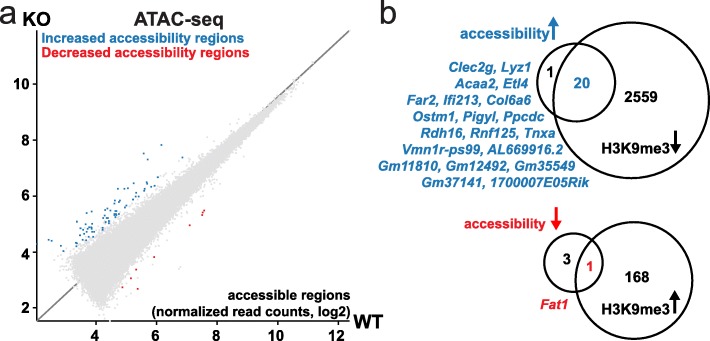
*Smarcad1*-KO changes chromatin accessibility in the small intestine. **a** The scatter plot of normalized read counts over ATAC-seq MACS peaks shows 84 differentially accessible regions (blue and red dots) as identified by EdgeR (*p*_adjust_ < 0.05, *n* = 3) on deletion of *Smarcad1* in the small intestinal epithelium (KO) compared to control (WT). For additional data and annotations, see Additional file 9: Table S8. **b** Comparison between genes with differential accessibility and genes with differential H3K9-trimethylation on *Smarcad1*-KO (ATAC-seq and ChIP-seq MACS peaks, annotation with closest gene ± 5 kbp, *n* = 3, see Additional file 6: Table S5, Additional file 9: Table S8). Of the genes that show increased accessibility and decreased H3K9me3, *Clec2g* and *Lyz1* are overexpressed on deletion of *Smarcad1* in the small intestinal epithelium (KO). There was no overlap between genes with increased accessibility and increased H3K9me3 nor with genes with decreased accessibility and decreased H3K9me3. The Venn diagrams are not drawn to scale

To test if deletion of *Smarcad1* affects accessibility to chromatin, we used the ATAC-seq approach on nuclei from small intestinal crypts [43]. This identified 84 sites that showed a significant change in accessibility, mostly an increase (76 sites increase, 8 decrease) (Fig. 5a, Additional file 9: Table S8). On gene level, we also observed a significant link between loss of accessibility and increase of H3K9me3 close to or over genes on *Smarcad1*-KO (Fig. 5b).

In summary, deletion of Smarcad1 leads to specific changes in histone H3K9me3 and this is linked to changes in chromatin accessibility and gene expression.

### Smarcad1 promotes colitis response

Because the gene expression analysis indicated that Smarcad1 is involved in regulating multiple genes linked to innate immunity and inflammatory processes, we tested the response of the intestine epithelium-specific knockout mice in the well-established dextran sodium sulfate (DSS)-induced colitis model, as DSS-mediated colitis is thought to depend critically on innate immunity [44]. DSS is a charged polymer that erodes the mucus layer in the colon when ingested through drinking water, which, in turn, exposes the colon epithelium directly to the microbial load of the colon lumen, eliciting an inflammatory response [45, 46]. Therefore, this model is considered especially valid for ulcerative colitis.

**Fig. 6:**
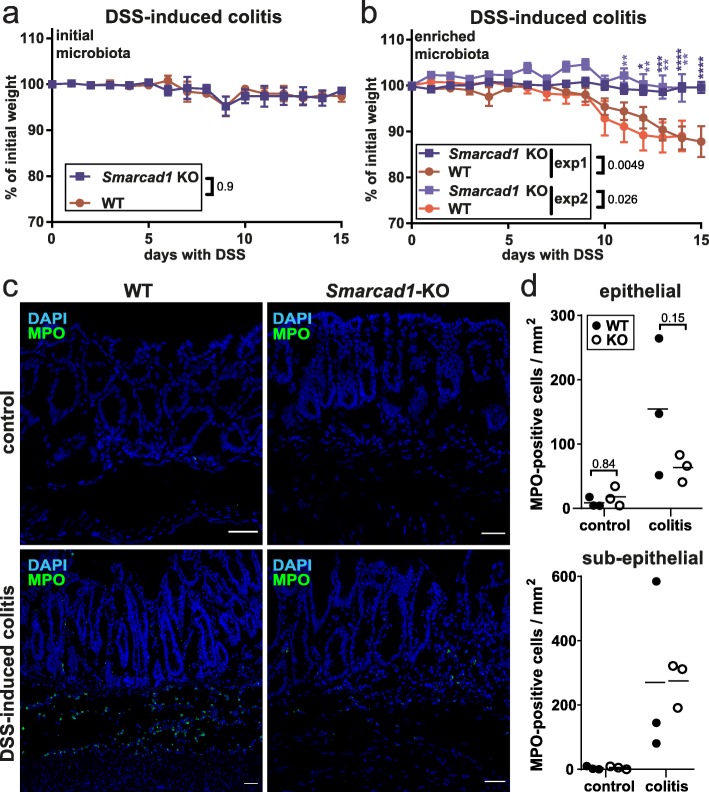
Smarcad1-dependent Colitis susceptibility. **a**, **b** 1% DSS-induced colitis phenotype in WT and *Smarcad1*-KO animals with initial (non-enriched) microbiota (**a**) and enriched microbiota (**b**, 2 independent experiments shown: b-1/b-2). Experiments **a** and b-1 were terminated after 15 days (*n* = 5 for WT/KO), and experiment b-2 after 14 days with one mouse culled after 10 days due to extensive weight loss (*n* = 8 for WT, *n* = 6 for KO). SEM indicated by error bars. Indicated *p* values determined by 2-way ANOVA with Holm-Sidak’s multiple comparisons test, performed separately for each experiment. *p < 0.05, **p < 0.01, ***p < 0.001, ****p < 0.0001. Full statistical results are listed in Additional file 2: Table S1. **c**, **d** Intestinal colon epithelium localization (**c**) and quantitation (**d**) of MPO-positive cells (green, neutrophil/lymphocyte-marker) by IF staining and nuclear counterstaining with DAPI (blue) shown as maximum intensity projections. The 14-day colitis induction with enriched microbiota. Representative replicates, full image set, and negative staining controls shown in Additional file 1: Fig. S7. Scale bars, 40 μm. WT, wild type; KO, Vil-cre-mediated tissue-specific knockout of Smarcad1. **d** Epithelial and sub-epithelial number of MPO-positive cells per imaged area, quantified from the full image set shown in Additional file 1: Fig. S7 (3 biological replicates for WT/KO, 2–4 technical replicates each). Indicated *p* values determined by 2-way ANOVA with Holm-Sidak’s multiple comparisons test, performed separately for each epithelial/sub-epithelial localization. Full statistical results are listed in Additional file 2: Table S1

**Fig. 7:**
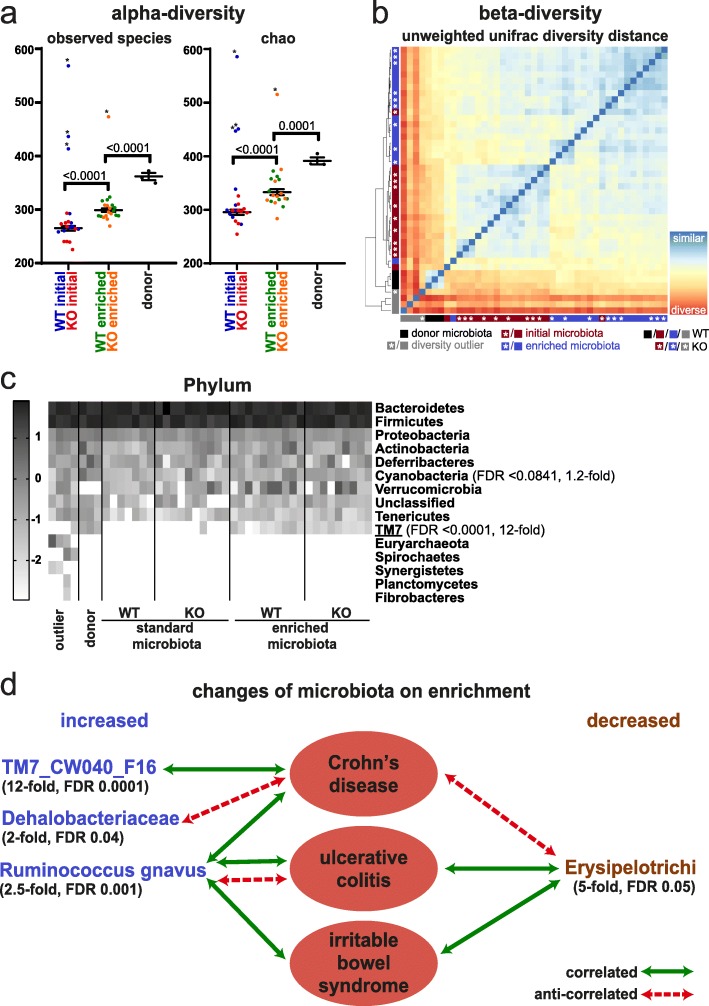
Microbiome and *Smarcad1*-dependent colitis susceptibility. **a** Alpha-diversity plots before and after microbiome enrichment, as well as donor microbiota, shown as observed species number (left) and chao-quantitation (right). Outliers (*) were identified with the ROUT algorithm in GraphPad Prism with the Q-cutoff set to 0.1%. Outliers were omitted for the following statistical analysis by 1-way ANOVA with Holm-Sidak’s multiple comparison test, *p* values indicated. SEM indicated by error bars. Full statistical results are listed in Additional file 2: Table S1. **b** Beta-diversity plot based on unweighted unifrac diversity distance (phylogenetic distance analysis of detected OTUs). Outliers previously detected based on alpha-diversity are indicated in gray. **c** Heat map of log10 transformed OTU abundance at the phylum level identifies TM7 as a phylum transferred on microbiota enrichment; see Additional file 1: Fig. S8 for other phylogenetic levels. Phylogenetic terms significantly different between initial and enriched microbiota (FDR < 0.1, Wilcoxon test, *n* = 20, outliers not excluded) are indicated with FDR and fold changes (enriched/initial). Terms shown in **d** are underlined. **d** Taxa substantially changed on microbiota enrichment (> 2-fold change, Wilcoxon test FDR < 0.05, *n* = 20, outliers not excluded) are indicated with FDR and fold changes between enriched and initial microbiota groups. Disease associations shown represent one or more previous studies in feces/colon biopsies from humans or mouse [49–64]. Where the cited studies have shown contradicting interactions, the predominant interactions are indicated

It is well established that the DSS colitis response depends on the composition of the microbiota, and it has been demonstrated that the microbiome of many mouse facilities lacks complexity [47, 48]. The latter is also true for the microbiome of the Babraham Institute mouse facility, as 1% DSS in the drinking water did not elicit a colitis response as seen by lack of weight loss (Fig. 6a, Additional file 1: Fig. S6a, c, Additional file 10: Table S9). Therefore, we decided to enrich our mice with microbiome from the mouse facility of the University of York that we knew had a strong colitis response, despite being specific pathogen free (SPF). We cohoused both sets of mice (control and intestine-specific Smarcad1-KO mice from Babraham) and York mice in a ventilated cabinet for 2 weeks, to allow for substantial transfer of microbiota. We profiled the microbiomes of the control and KO mice before and after cohousing, as well as donor microbiomes by 16S RNA amplicon sequencing. This showed no significant differences of the control and KO mice before or after this exposure, indicating that the deletion of Smarcad1 does not affect microbiome composition in a major way (Fig. 7b). In contrast to this, the donor microbiome was clearly distinct and more complex from the recipient microbiome (Fig. 7a–c, Additional file 1: Fig. S8). Furthermore, we detected transfer of specific microbial species (Fig. 7c, Additional file 1: Fig. S8c-g). Our analysis highlights at the phylum level the significant transfer of TM7 (Fig. 7c, d, see the “Discussion” section). At the family level, we see transfer of *Dehalobacteriaceae* and at the species level *Ruminococcus gnavus*. This analysis shows also significant loss of class *Erysipelotrichi* on transfer in the recipient animals.

Next, we repeated the colitis experiments with the microbiome-enriched control and *Smarcad1*-KO mice.

We found that the microbiome-enriched control mice reacted with a clear colitis response to DSS, developing soft stool and, thereafter, losing significant amount of weight. Remarkably, the intestine-specific *Smarcad1*-KO mice did not exhibit this phenotype (Fig. 6b, Additional file 1: Fig. S6b, d-f, Additional file 10: Table S9).

It is known that DSS-mediated colitis is associated with focal invasion of neutrophils and monocytes into the colon epithelium [44]. We did observe infiltration of these cell types in the DSS-treated wild type mice, as shown by anti-myeloperoxidase (MPO) staining (Fig. 6c, d, Additional file 1: Fig. S7). Consistent with a reduced colitis response, this occurred to a lesser extent in the *Smarcad1*-KO mice (Fig. 6c, d, Additional file 1: Fig. S7).

**Fig. 8:**
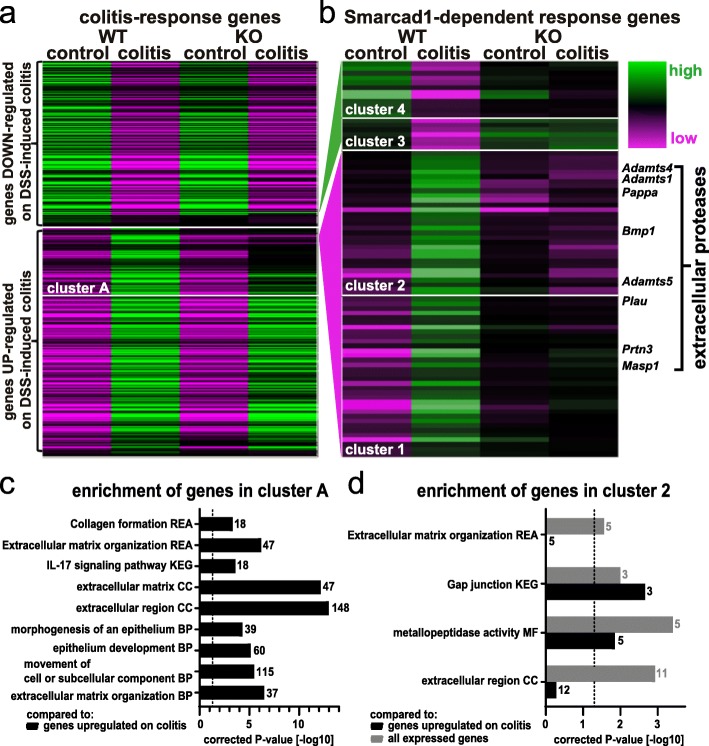
Smarcad1-dependent transcription response. Changes of gene expression on DSS-induced colitis in microbiota-enriched animals. **a** WT/*Smarcad1*-KO heat map of all differentially expressed genes (DEG) between control and DSS-induced colitis in colon epithelium samples (WT-colitis vs. WT-control, DESeq2, FDR < 0.05, Additional file 13: Table S12). Cluster A (Additional file 14: Table S13) contains genes not upregulated on colitis in *Smarcad1*-KO colon to the same extent as in WT. **b** DEG showing a diminished colitis response on Smarcad1 presence. Classified in 4 clusters. A full list of Smarcad1-dependent response genes in the indicated clusters is attached with functional annotations in Additional file 15: Table S14. The enriched genes annotated as extracellular proteases are labeled. **c**, **d** Enrichment of selected terms detected by gene ontology analysis. BP, biological process; CC, cellular component; MF, molecular function; KEGG, KEGG biological pathways; REA, reactome. Dashed line indicates significance threshold corrected *p* = 0.05. Gene number annotated in the cluster with a specific term is indicated next to each bar. **c** Cluster A (Additional file 14: Table S13) vs. genes upregulated on colitis in WT (Additional file 13: Table S12, marked UP). **d** Cluster 2 versus genes expressed in the colon (Additional file 16: Table S15) shown in gray and versus genes upregulated on colitis in WT (Additional file 13: Table S12) shown in black. Full gene ontology enrichment analysis is listed in Additional file 17: Table S16 and Additional file 18: Table S17

To explore this on a molecular level, we extracted mRNA from colon tissue of untreated mice and from mice after DSS treatment and performed transcriptome analysis by RNA-seq (Additional file 1: Fig. S9, Additional file 11: Table S10, Additional file 12: Table S11). We identified 3261 DEG on colitis induction in WT (wild type) mice (DESeq2 test, cutoff false discovery rate (FDR) < 0.05, Additional file 13: Table S12), and gene ontology analysis confirms a strong link of these genes to inflammatory responses (Additional file 1: Fig. S6g). Most of these genes respond in the similar way to colitis on *Smarcad1*-KO (Fig. 8a). However, a subset of genes shows incomplete upregulation on colitis induction in Smarcad1-KO mice. We identified these genes as cluster A (572 genes, see Additional file 14: Table S13). Upon gene ontology analysis comparing cluster A to the gene list upregulated on colitis in WT (see annotation in Additional file 13: Table S12), we identified a number of enriched terms (g:profiler, full parameters and enriched terms: Additional file 19: Table S18). The most biologically meaningful terms, shown in Fig. 8c, indicate potentially *Smarcad1*-dependent pathways in the complex colitis response.

A finer resolved hierarchical clustering was performed to detect genes with complete or near complete loss of expression changes on colitis in *Smarcad1*-KO. These genes were identified as clusters 1/2 (normally upregulated on colitis in WT) and 3/4 (normally downregulated on colitis), with a total of 84 genes (Fig. 8b and listed with relevant annotations in Additional file 15: Table S14). Gene ontology (GO) analysis of clusters 3/4 did not yield any enriched annotations, probably due to the small size of these clusters (g:profiler, cutoff *p* < 0.05, Additional file 20: Table S19). GO analysis of cluster 2, containing genes with complete loss of Smarcad1-dependent upregulation on colitis, yielded several enrichment terms (Fig. 8d, Additional file 17: Table S16, Additional file 18: Table S17). This includes the significantly enriched group of extracellular protease encoding genes (also indicated in Fig. 8b). Stainings for Adamts1, Adamts5, and Bmp1 showed similar distribution patterns as the MPO-staining, indicating that these proteases are contributed not by the colon epithelium, but by invading neutrophils/monocytes on colitis induction (Additional file 1: Fig. S7). The GO analysis also highlights a potential role of Smarcad1 in the IL-17 pathway in colitis. The transcriptome-based analysis of the colitis response illustrated the upregulation or shutdown of expression of many genes upon DSS treatment and shows that this transcriptional response was subdued for many genes in the intestine-specific *Smarcad1*-KO mice.

## Discussion

Previous work has indicated an important role of Smarcad1 and its homologs in the maintenance of heterochromatin, especially during or following the DNA replication process [10, 11] and the silencing of endogenous retroviruses in embryonic stem cells [65], but the importance of this role in a tissue context was not clear. We found that this factor is highly expressed in the crypt of the small intestine, which is the zone of cell proliferation, consistent with a role during chromatin replication. Here, we did not find evidence that Smarcad1 affected global H3K9me3 levels, as we have previously shown in cultured cells. However, on genome-wide analysis, we found significant changes of H3K9me3 upon *Smarcad1*-KO and many of these changes are a loss of H3K9me3 over a defined region, close to or in the vicinity of genes, including many upregulated genes. We found a significant link between these changes and alterations in gene expression, especially in the small intestinal epithelium, and several of the affected genes are linked to innate immunity processes.

We observed a notable difference in the number of sites where H3K9me3 decreases between the small intestine and colon epithelia, possibly linked to the histological differences in the cells we isolated for the analysis. We isolated crypts of both small intestine and colon tissue for the ChIP-seq analysis. Colon crypts contain proportionally more differentiated cells compared to small intestine crypts. Future analysis should unravel the role of Smarcad1 in H3K9me3 establishment and maintenance during differentiation.

Unlike the study from Sachs et al. that document Smarcad1 binding by ChIP-seq [65], we have not been able to produce a Smarcad1 ChIP-seq profile employing same antibodies and protocols. This may relate to the much lower expression of Smarcad1 in somatic type cells compared to embryonic stem cells, as shown by the Sachs et al. study. Furthermore, the predominant localization of Smarcad1 in the stem and proliferative compartment of the intestinal epithelium is consistent with a global rather than locus-specific role, e.g., maintenance of heterochromatin through replication [10].

Interestingly, *Smarcad1*-KO led to an increase of H3K9me3 over broader regions within the coding regions of a number of genes, and in some cases, this was linked to decreased expression. It is possible that Smarcad1 has a role during transcription elongation of a gene subset. A role in transcription elongation has been suggested for the fission yeast Smarcad1 homolog Fft3 [66]. However, as we find such changes only in select genes, this activity appears to be gene specific.

We only observed few changes in gene accessibility by our ATAC-seq approach. While this may be due to technical limitations, it might reflect the biology of the intestinal epithelium. In this context, it is interesting that a previous study found that changes in gene expression during intestinal cell maturation do not involve dramatic changes in chromatin accessibility [67].

We found that intestine epithelium-specific *Smarcad1*-KO protects the mice from DSS-induced colitis, reducing the response in terms of weight loss, disease activity index, MPO^+^ cell recruitment, and gene expression response. This observation is striking, as a majority of gene deletions would be expected to lead to increased colitis susceptibility. However, there is precedence for such an observation. For example, monoallelic deletion of the non-muscle-myosin-II (NMII) heavy chain *My9* gene alleviates DSS-induced colon crypt damage and colitis, possibly by promoting intestinal stem cell turnover [68]. We did not find evidence that deletion of *Smarcad1* affects intestinal cell turnover or epithelial barrier function, but rather, we found that it leads to changes in gene expression that are consistent with a protective effect. In the steady-state condition, we found that *Smarcad1*-KO promoted expression of several genes linked to innate immunity, such as Toll-like receptor *Tlr4*, anti-bacterial peptides, and other factors controlling inflammatory responses such as *Lyz1*. One gene that is highly overexpressed upon *Smarcad*1-KO in steady state and colitis is *Mt1*, coding for metallothionein. Metallothionein has been reported to protect against colitis in mouse models, but its role in this process requires further investigation [27, 69–73]. Another gene that is upregulated upon Smarcad1 deletion codes for Bambi. Bambi is a TGF-beta decoy receptor that dampens or blocks the activity of this cytokine and thus controls inflammatory responses [30, 74]. TGF-beta is involved in inflammatory responses, including in colitis [75]. This suggests that Smarcad1 is normally involved in a pathway that controls an innate immunity response, and upon its deletion, the intestinal epithelium may already be primed to deal with a microbial challenge during the DSS treatment. On DSS treatment, the deletion of *Smarcad1* leads to specific changes in gene expression consistent with the reduced colitis response. Together, our data suggest that this chromatin remodeling factor orchestrates the expression of genes involved in an inflammatory response in the gut. As we made these observations with an intestinal epithelium-specific deletion of *Smarcad1*, our observations underscore the importance of the intestinal epithelial tissue and innate immunity-linked processes in mediating a colitis response.

We identified several members of the gut microbiome that are responsible for a robust colitis response in a *Smarcad1*-dependent way. These are candidates for promoting colitis disease progression during the DSS treatment, requiring the presence of Smarcad1 in the intestinal epithelium. Interestingly, these are members of a healthy SPF microbiome and are not normally associated with disease without additional challenge. Standing out among these in terms of consistency and fold increase are members of the *TM7* (also called Saccharibacteria) phylum, recently described and poorly understood Gram-positive bacteria. Interestingly, the one TM7 member that has been cultivated so far is an epibiont and parasite on another bacterial species, affecting the hosts’ interaction with the human immune system [76]. A potential link between *TM7* strains and Crohn’s disease has been previously described [49]. *TM7* levels are regulated by the inflammasome [77] and have been linked to inflammatory activity of the microbiome in aged mice [78] and changes in the mucus layer barrier function of the distal colon [79]. Another increased species is *Ruminococcus gnavus*, a member of the class *Clostridia*. Increased levels of *R. gnavus* have been linked to intestinal inflammatory diseases [50–53, 80]. *R. gnavus* is a mucolytic bacterium which alters mucus protective function [54, 81]. We also see a loss of members of the class *Erysipelotrichi*, which promote barrier function of the colon epithelium [79]. Overall, the changes we detect in our enriched microbiota are consistent with the greater colitogenic effect. Whether it is a single species, such as *TM7*, that drives this effect or the combination of several remains to be elucidated.

## Conclusions

Our study demonstrates the critical role of chromatin dynamics in intestinal epithelial cells in host-microbiome interactions, regulating the colitis response. We uncover the role of a highly conserved chromatin remodeling factor in this process and show that it operates by affecting the repressive H3K9me3 histone modification over genes that are involved. In addition to Smarcad1, we identify candidate bacterial species crucial for the phenotype severity of colitis, highlighting their potential as targets for pharmacological and probiotic treatment of intestinal inflammatory diseases.

## Methods

### Mice

All mice were C57BL/6 background, males, and kept in specific opportunistic pathogen free (SOPF) conditions at the Babraham Institute transgenic facility and fed CRM (P) VP diet (Special Diet Services) ad libitum. Animals were sacrificed by CO_2_ asphyxiation followed by cervical dislocation.

### Conditional deletion of *Smarcad1*

We generated mice with *loxP* sites integrated in the introns between exons 11 and 12 and between exons 14 and 15 of *Smarcad1* through recombineering [82]. Details regarding the construction of a Smarcad1 targeting vector and generating transgenic mice are available on request. BAC clone RP23-331E23, which completely spans the *Smarcad1* gene, constructed by the laboratory of Pieter de Jong at Roswell Park Cancer Institute was obtained from MRC Geneservice. BAC DNA was electroporated into *E. coli* EL350. Positive clones were selected with 12.5 μg/ml chloramphenicol. The construction of retrieval vector was essentially as described in [82]. In brief, two pairs of PCR primers denoted as A, B and Y, Z were designed. These amplify ~ 500 bp segments located 14.6 kbp apart within the Smarcad1 gene. Separate PCRs were carried out using A + B and Y + Z oligos on BAC 331E23. The purified AB product was cleaved with Not1/HindIII and the YZ product with HindIII/Spe1. After digestion, AB and YZ fragments were ligated into Not1/Spe1 cut vector PL253 and cloned in *E. coli*. The retrieval plasmid was linearized with HindIII and transformed into EL350 cells containing the BAC, and cells containing the desired recombinant molecule were selected on ampicillin. This resulted in cells where BAC DNA was successfully retrieved into PL253, called pGRSB (**G**ap **R**epaired **S**marcad **B**AC).

In order to introduce a floxed Neo cassette, the Neo cassette in PL452 was amplified by PCR with 300 bp arms. Two pairs of PCR primers were used: CD and EF. Primers E and F contained BamHI and NotI sites in their respective tails; C and D contained SalI and EcoRI sites. PCR product EF was purified, digested with BamH1/NotI, and ligated into BamHI/NotI cut PL452. Transformed *E. coli* were selected on 50 μg/ml ampicillin, and the obtained plasmid, termed PL452EF, was digested with SalI/EcoRI and ligated to SalI/EcoRI digested PCR product CD. The resulting plasmid was termed PL452CDEF. Next, the Neo cassette with two flanking regions of *Smarcad1* was isolated by digestion of PL452CDEF with SalI/NotI. This yielded a 2.6-Kb fragment which was purified and recombined into the Gap-retrieved BAC. The Neo cassette was electroporated and recombined in EL350 cells containing the retrieved BAC, and selection was with kanamycin resulting in plasmid pGRSB5’Neo. The Neo cassette was now excised by electroporation into cells with 0.1% arabinose-induced Cre recombinase and selected on 50 μg/ml ampicillin or 12.5 μg/ml kanamycin. Plasmid from colonies which grew on ampicillin but not kanamycin was digested with BamHI and PCR amplified with oligos derived from PL452 sequences flanking the SalI and NotI sites, respectively. Two clones, termed pGRSB5’loxP9 and pGRSB5’loxP11, were confirmed by sequencing to contain a single *loxP* site integrated at the correct location. Next, the downstream Neo cassette, derived from PL451 (containing FRT–PGK–EM7–NeobpA–FRT–loxP), was assembled. PCR primers G, H, I, and J containing recognition sites for SalI, HindII, BamHI, and NotI in their respective tails were synthesized. PCR product GH was digested with SalI/EcoRI and then ligated into SalI/EcoRI digested PL451 and transformed into *E. coli* Top10, digested with BamHI/NotI, and ligated to BamHI/NotI cut PCR product IJ. Ligations were transformed into Top10 cells yielding plasmid PL451-GHIJ. PL451-GHIJ was digested with NotI/SalI. The insert, comprising the PL451 Neo cassette flanked by Smarcad1 fragment GH and IJ, was now recombined into the targeting vectors pGRSB5’loxP 9 and 11, containing the upstream (left hand) *loxP* site with 50 μg/ml ampicillin selection. GH-Neo-IJ insert was electroporated into induced EL350 cells with kanamycin selection (12.5 μg/ml). The resulting plasmids PGRSB5’loxP3’Neo were re-transformed into NovaBlue with kanamycin selection (12.5 μg/ml). Digestion with BamH1 and sequencing using G and J oligos confirmed correct assembly of the final construct.

One hundred micrograms of pGRSB5’loxP3’neo was linearized with NotI and transformed into Bruce4 ES cells using electroporation by the Babraham Institute Gene Targeting facility. We mapped correct integration of the targeting construct by Southern blotting after digestion of genomic DNA with BamHI. We confirmed that the four correctly targeted clones contained a single integration of the targeting cassette, by cleaving DNAs with BglII followed by gel electrophoresis and Southern blotting. Two of the four positive ES cell clones, C: D3 and C: E6, were injected into C57BL/6 blastocysts. This gave rise to chimeras and ultimately two mouse lines where Smarcad1 had been targeted: CD3 and CE6. We found that on intestinal deletion of the exons by *Vil-cre*, both mouse lines overexpressed *Bglap3*. We decided to use the CE6 line for all further studies.

The *Vil-cre* allele was introduced through the Tg (Vil1-cre)997Gum mouse [24] (JAX stock #004586, The Jackson Laboratory). Genotyping PCR protocols are available on request. To isolate small intestinal stem cells, we crossed in the Lgr5-EGFP-IRES-creERT2 “knock-in” allele (Lgr5-EGFP-IRES-creERT2 mice, C57BL/6 J background, obtained from Jackson Laboratory [83]).

### DSS-induced colitis

Within each experiment, the cohorts were age matched and, as much as possible, litter matched. The order of samples from cohorts was mixed on collection. No samples were excluded from the analysis. No blinding was conducted. Mice were acclimatized to the experimental setup in ventilated cabinets (scantainers) 2 weeks prior to DSS administration. Enriched microbiota was provided from this time point by bedding transfer and co-ventilation with the donor C57BL/6 females obtained from the University of York SPF facility. One percent dextran sulfate sodium salt (Sigma Aldrich 42867) was continuously administered with drinking water and exchanged every 3 days. Animal health status and weight were recorded daily.

### Barrier permeability assay

FITC-dextran flux measurements were performed as described [84]. Blood was collected from tail veins.

### Epithelial cell isolation for RNA-seq and ChIP-seq

Small intestines were dissected as described [85]. Colons were dissected by the same protocol with extended PBS-EDTA incubation (30 min) and crypt shaking extraction (5 min). Whole crypt epithelium RNA was isolated and ChIP-material processed at this stage. Single cell material was derived from intestinal crypts by digestion with Dispase II (Sigma D4693, 0.05 mg/ml), DNase (Qiagen 79254, 20 Kunitz U/ml), and Collagenase (Sigma C7657, 0.15 mg/ml), while villus single cells were derived without digestion, by vigorous pipetting. Small intestinal villus- and colon crypt-derived cells were stained in 2% fetal bovine serum (FBS)/phosphate buffered saline (PBS) with 1:200 APC-conjugated Epcam antibody (CD326, eBioscience 17-5791-82), 1:1000 AF488-conjugated CD45 antibody (Biolegend 103122), and 1:1000 AF488-conjugated CD31 antibody (Biolegend 102414) for 15 min at room temperature, washed 3 times and resuspended in 2% FBS/PBS with 3.2 μg/ml ROCK-inhibitor (Y-27632, Sigma). All cell preparations were stained with 4′,6-diamidino-2-phenylindole (DAPI) prior to sorting. Epcam^high^ CD31^−^ CD45^−^ DAPI^−^ cells were sorted on BD Aria III SORP cell sorter, 100 μM nozzle. RNA from ISC (GFP^high^ DAPI^−^) and transit amplifying cells (TA, GFP^medium^ DAPI^−^) was isolated from single cell crypt suspensions of Lgr5-GFP^+^mice and processed as above except the antibody staining step (Additional file 1: Fig. S10). 1000–80,000 ISC, 1000–135,000 TA, 100,000 AE, and 75,000–100,000 colon epithelial cells (CO) were sorted per sample into RLT buffer for subsequent RNA isolation with the RNeasy Micro kit (Qiagen 74004) with on-column DNA digestion.

### RNA-seq ISC, TA, AE, and CO

Libraries were generated from 2 to 10 ng total RNA with RNA integrity number (RIN) 6.2–9.1 as input according to NEB Ultra II Directional RNA Library Preparation Kit for Illumina (E7760), Poly(A) mRNA magnetic isolation module (E7490), and multiplex oligos (NEB E7335, E7500) manuals with the following modifications: RNA was fragmented for 20 min, adaptor stock was diluted 150-fold, and 15 cycles were used in the PCR-amplification step. SPRI select beads were substituted with Seramag Speedbeads (Thermo scientific 65152105050250) for size selection steps. Seramag beads were washed with TE buffer and resuspended in 50 volumes of PEG 8000 (Sigma 1546605) with 2.5 M NaCl, 10 mM Tris-Cl pH 8.0, 1 mM EDTA, and 0.05% Tween 20. PEG 8000 amounts used were 10 and 12% final PEG concentrations on sample addition. Size selection steps were performed at room temperature, adding the sample topped up to 100 μl with nuclease-free water to 80 μl of bead suspension, followed by resuspension by pipetting, incubation for 10 min, and magnetic pelleting. After removal of supernatant (SN), the beads were washed twice with 80% ethanol and moderately dried before elution in TE buffer. The size selection after second strand DNA synthesis was performed with 12% final PEG concentration, and the remaining size selections with 10% PEG. After PCR amplification, the size selection was performed twice (10% PEG). Libraries were sequenced on an Illumina HiSeq2500 as HiSeq 50 bp single-end reads.

### Colon epithelium isolation for RNA-seq after DSS treatment or enrichment of microbiota

The central 1/3 of the colon was cut open longitudinally, washed in PBS to remove feces, and snap frozen in liquid nitrogen. Twenty-five to 100 mg of frozen tissue was ground to powder with dry ice. After addition of 1 ml TRIzol, the sample was collected and incubated 5 min at RT. Two hundred microliters of chloroform was added prior vortexing and centrifugation 20 min at 16,000×*g*, 4 °C. The upper phase was transferred to 500 μl isopropanol, vortexed and incubated 15 min at RT before centrifuging 10 min at 16,000 x *g*, 4 °C. To deplete DSS, after discarding the SN, LiCl-precipitation was performed [86] at 4 °C by dissolving the pellet in 120 μl H_2_O and addition of 80 μl 2 M LiCl and 2 h incubation prior to centrifugation for 30 min at 14,000 x *g*. LiCl-precipitation was repeated once more and the pellet dissolved in 200 μl H_2_O. Next, the RNA was precipitated substituting LiCl with 20 μl 3 M NaAc, pH 5.2 and 400 μl 100% EtOH, with 30 min incubation at − 20 °C. After centrifugation, the pellet was washed once in 70% EtOH before resuspending in cold H_2_O.

### Whole tissue small intestine isolation for RNA-seq

RNA from small intestine whole tissue was isolated as described above for whole colon tissue, without LiCl precipitation steps.

### RNA-seq colon epithelium after DSS treatment and enrichment of microbiota

At least 1 μg total RNA (RIN 7.2–8.5) per sample were sequenced by BGI Hong Kong on the BGISEQ-500 platform as 100 bp paired-end reads, yielding fastq files filtered for low-quality, N-rich, or adaptor-polluted reads.

### Western blotting

Small intestinal crypts and villi, as well as colonic epithelium, were extracted as described above including the shaking extraction. The epithelium was then pelleted 10 min at 500×*g*, resuspended in Laemmli 2x lysis buffer supplemented with 5% Beta-Mercaptoethanol and boiled for 1 min. Samples were briefly sonicated to reduce viscosity.

Anti-Smarcad1 (Sigma HPA016737, 0.3 μg/ml), anti-H3 (Abcam 1791, 0.025 μg/ml), anti-Osteocalcin (SantaCruz 365797, 4 μg/ml), anti-H3K9me2 (Abcam 1220, 0.18 μg/ml), and anti-H3K9me3 (Abcam 8898, 0.2 μg/ml) antibodies were used for Western blot with BSA blocking, Tris-buffered saline (TBS) 0.05% Tween-20 (TBS-T) washing buffer, and enhanced chemiluminescence (ECL) detected on x-ray film. Protein ladder was from Thermo Scientific #26616. Uncropped Western blots are shown in Additional file 1: Fig. S11.

### Immunofluorescent staining

Smarcad1 immunofluorescent (IF) staining was performed as described in [87] with the following antibodies: primary anti-Smarcad1 (Sigma-Aldrich HPA016737, 1.5 μg/ml) and secondary anti-rabbit AlexaFluor 568 (Invitrogen A11036, 2 μg/ml). MPO, Adamts1, Adamts5, and Bmp1 IF stainings were performed, and MPO-positive cells quantified as described in [88] with the following antibodies: primary anti-MPO (R&D AF3667, 5 μg/ml), primary anti-Adamts1 (Abcam ab39194, 1 μg/ml), primary anti-Adamts5 (Abcam ab246975, 0.5 μg/ml), primary anti-Bmp1 (Abcam ab38953, 2 μg/ml), and secondary anti-goat AlexaFluor 488 (Invitrogen A11055, 10 μg/ml) and anti-rabbit AlexaFluor 568 (Invitrogen A11036, 20 μg/ml). Confocal imaging was performed on a Zeiss 780 confocal microscope with a × 20 Plan Apo air objective at optimal resolution settings with 2× line averaging. Contrast enhancement with minor pixel saturation was performed with FIJI [89]. MPO-staining was performed as 2–4 technical replicates (different intestinal positions within the same sample) that were measured and averaged per sample. The following are the average total imaged per sample: 0.47 mm^2^ epithelial area and 0.30 mm^2^ sub-epithelial area. Imaging areas were measured with FIJI, and positive cells counted manually after background correction.

### 5-Ethynyl-2′-deoxyuridine (EdU) labeling

One hundred micrograms of EdU (Invitrogen, A10044) in 200 μl PBS per mouse was injected intraperitoneally (animal weight 29–36 g). Mice were sacrificed and processed after 24 h. The intestine was extracted, flushed with PBS at 4 °C, and fixed in 4% formaldehyde (FA)/PBS solution for 24 h at 4 °C. After cryopreservation in 30% sucrose for 24 h at 4 °C, the samples were frozen in Cryomatrix (ThermoFisher 6769006). Frozen blocks were cut with the Leica CM1860 cryotome to 10 μm sections onto Superfrost Plus slides (ThermoFisher 4951PLUS4). Before staining, slides were dried for 15 min at room temperature and refixed with 4% FA/PBS. Samples were permeabilized in 1% TritonX-100/PBS at RT for 20 min, washed 3× for 5 min in PBS, and incubated in blocking buffer (1% TritonX-100, 5% FBS, 2% BSA) for > 1 h. Click-iT reaction was performed according to the manufacturer’s instructions (Life Technologies, Click-iT Plus EdU imaging kit C10637). 0.5 ml Click-iT reaction mix were incubated on the sample for 30 min at RT protected from light and washed with 3% BSA/PBS before mounting with Vectashield (Vector, H-1200, with DAPI). Sections were imaged with the Zeiss780 confocal microscope, × 20 air objective at optimal resolution settings and 2× line averaging. FIJI [89] and Excel were used to analyze EdU signal distance to crypt bottom (see Additional file 24: Table S23, Additional file 25: Protocol S1).

### Small intestinal organoid culture and RNA-seq on organoids

Small intestinal crypts were derived from mice where Smarcad1 had been deleted in oocyte development using ZP3-cre and control litter mates, using a slightly modified protocol as described [87]. Isolation of small intestinal crypts for organoid culture was performed using a modified version of a previously described protocol [90]. Small intestines were collected and opened longitudinally. The villus content was removed by scraping the intestine with a coverslip and by shaking the samples vigorously in cold DPBS (Dulbecco’s phosphate-buffered saline). The intestines were then incubated at 4 °C in DPBS containing 2 mM EDTA for 30 min and in DPBS with 5 mM EDTA for another 30 min, shaking the samples in between to separate the crypts from the connective tissue. Large material was removed by passing the SN through a 40-μm cell strainer. One hundred to 200 small intestinal crypts were suspended in cold liquid Matrigel and incubated at 37 °C for 15 min for Matrigel gelling. Complete ENR medium containing advanced DMEM/F12 (Sigma), 2 mM Glutamax (Invitrogen), 10 mM HEPES (Gibco), 100 U/ml penicillin/streptomycin (Invitrogen), 1 mM *N*-acetyl-cystein (Sigma), B27 supplement (Invitrogen), N2 supplement (Invitrogen), 50 ng/ml mouse EGF (Peprotech), 100 ng/ml mouse Noggin (Peprotech), and 10% human R-spondin-1-conditioned medium from R-spondin-1-transfected HEK 293 T cells (HA-R-Spondin1-Fc 293 T stably transfected cell line, expressing murine Rspo1 with an N-terminal HA epitope tag and fused to a C-terminal murine IgG2a Fc fragment, obtained from Dr. Calvin Kuo, details on request) was added to the cultures. Media were replaced every 2 days. For the organoid passages, after 7 days of culture, Matrigel was mechanically disrupted with a P1000 pipette tip and cold media were added to help it to liquify. The organoids were broken into small fragments by pipetting them up and down several times using a P200 pipette. Organoid fragments were pelleted using centrifugation at 465×*g* for 5 min at 4 °C to wash away the old Matrigel and debris. The organoid fragments were re-suspended in cold liquid Matrigel (50% Matrigel and 50% complete ENR medium) and reseeded as described before. After 5 days, organoids were collected for RNA extraction. For RNA-seq, total RNA was extracted from ~ 600 organoids using TRIzol reagent (Invitrogen) and the DNA-free™ DNA Removal kit (ThermoFisher) following the manufacturers’ specifications. Poly(A)+ RNA was selected using NEBNext Oligo d(T)25 beads (NEB). Libraries were prepared with NEBNext Ultra directional RNA library Prep kit for Illumina (Cat. EZ420S) in combination with NEBNext Poly(A) mRNA Magnetic Isolation Module. Five hundred nanograms of RNA was used with the fragmentation step at 94 °C for 16 min. PCR amplification was performed using the KAPA HiFi HotStart PCR kit, 14 cycles.

### ChIP-seq

Small intestinal and colon crypts were resuspended in 1 ml of 1% FA/PBS and fixed for 10 min at RT. The reaction was quenched with glycine at a final concentration of 0.125 M for 5 min. Cells were pelleted at 2400×*g*, 4 °C for 2 min and washed three times with 1 ml of PBS. Sonication was performed in 180 μl of lysis buffer (50 mM Tris-HCl, pH 8.0, 10 mM EDTA, 1% SDS, and protein inhibitors cocktail) in a water-cooled Bioruptor (Diagenode), high power, for 14 cycles with 30 s on and 30 s off. The sonicated chromatin was diluted in 810 μl of RIPA buffer containing 0.1% SDS and incubated overnight with protein A/G sepharose Dynabeads previously incubated with the specific antibody for ChIP (H3K9me2—ab1220, and H3K9me3—ab8898 Abcam) for 2 h. For the inputs, 3% of the volume for each sample was used. Following incubation, the material was washed 2 times with RIPA low salt buffer (20 mM Tris-HCl, pH 8.0, 2 mM EDTA, 150 mM NaCl, 1% Triton-X-100, 0.1% SDS) and 2 times with RIPA buffer high salt (containing 500 mM NaCl) and once with TE buffer (10 mM Tris-HCl, 1 mM EDTA). Samples were diluted in 200 μl of elution buffer (20 mM Tris-HCl pH 7.5, 5 mM EDTA, and 50 mM NaCl) and treated with 2 μl of RNase (20 mg/ml) for 30 min at 37 °C. After this treatment, samples were incubated with proteinase K for 2 h at 65 °C at 300 rpm. The DNA was extracted with phenol-chloroform-isoamyl alcohol. Library preparation was performed with 5 ng DNA with the NEBNext Ultra II DNA Library Prep for Illumina, according to the manufacturer’s instructions and sequenced on the Illumina HiSeq2500 platform, 100 bp pair-end.

### ATAC-seq

Mice were killed, and small intestines extracted, immediately flushed with ice cold PBS, cut open longitudinally, and rinsed 3× for 10 s with HBSS (Hank’s balanced salt solution) without Ca^2+^/Mg^2+^. The intestines were incubated 10 min in 30 mM EDTA/HBSS on ice, then shaken vigorously by hand for 5 s and the SN removed. They were incubated a further 20 min in 30 mM EDTA/HBSS on ice and then shaken vigorously for 5 min. Villi and mucus were removed by dripping the material through a 100- then 70-μm strainer. Crypts were pelleted at 170×*g* at 4 °C for 10 min and SN removed. The crypts were washed with 10 ml ice cold PBS, re-pelleted, and SN removed, twice. Crypts were resuspended in 2 ml TrypLE Express with 10 μM Y-27632 and 0.5 mM *N*-acetylcysteine, pipetted carefully with a 1-ml pipet, and dissociated at RT and monitored by microscopy. The suspension was then topped up with 20 ml 10% FBS/PBS, and the cells were filtered through a 40-μm strainer. The cell suspension was then pelleted at 465×*g*, 4 °C for 5 min and resuspended in 5 ml ice cold HBSS, twice. The cells were re-suspended in 2 ml 1% Triton-X-100 containing N-buffer (15 mM HEPES, pH 7.5, 10% sucrose, 60 mM KCl, 15 mM NaCl, 0.5 mM EGTA, 0.2 mM PMSF, 1× Complete™ (Roche) protease inhibitor, 50 mM sodium butyrate) and incubated 15 min on ice. This mix was then overlaid on a 5-ml sucrose cushion (30% sucrose in N-buffer), and nuclei were pelleted for 15 min at 1300×*g*, 4 °C. Nuclei were taken up in 100 μl ice cold nuclei storage (25 mM Tris-HCl, pH 7.5, 100 mM potassium acetate, 10 mM MgCl_2_, 2 mM Spermidine) and counted. ~ 50,000 cells were used for each ATAC-seq library as described [43] using the Nextera kit from Illumina with TruSeq primers. Libraries were sequenced 50 bp, paired end.

### Bioinformatic analysis of ChIP-seq and ATAC-seq data

Reads were adaptor trimmed with Trim Galore (v0.4.4 for ChIP-seq and v0.4.1 for ATAC-seq) and mapped to the mouse reference genome GRCm38/mm10 with Bowtie 2 (v2.3.2 for ChIP-seq and v2.2.5 for ATAC-seq). We used SeqMonk version 1.44.0 for the bioinformatic analysis of ChIP-seq and ATAC-seq data. For H3K9me2 and H3K9me3 analysis, we imported paired-end reads as .bam files with the minimal mapping quality cutoff “20” and maximal distance 1500 bp cutoff, and duplicates removed on import. We used MACS peak finder integrated in SeqMonk using all ChIP samples and for “input” the input libraries from control and KO, fragment size 300, significance threshold 1 × 10^−5^. We used EdgeR, embedded in SeqMonk to identify peaks that change in KO versus control (WT, *p* < 0.05 after Benjamini-Hochberg multiple testing correction). To generate browser shots, we generated running window probes of 200 bp with 100 bp overlap and smoothed these further over 5 probes.

For ATAC-seq, we imported .bam files with 1000 bp cutoff, duplicates removed, quality cutoff “20,” and identified MACS peaks using SeqMonk with default settings. We used EdgeR embedded in SeqMonk with significance cutoff *p* < 0.05 after Benjamini-Hochberg multiple testing correction to identify peaks that change in KO compared to control (WT).

### Microbiome identification

Feces were collected from microbiota donors and WT/*Smarcad1*-KO littermates after 28 days of cohousing (as described above except the DSS supplementation) with donor mice, which corresponds to a full DSS-induced colitis experiment timeframe. Two fecal pellets/mouse were processed by BGI Hong Kong as 250 bp paired-end read (Illumina) and analysis via the 16S rDNA-amplicon pipeline. Low-quality and adaptor-polluted reads were removed prior to paired-end merging to tags. Tags were assigned to operational taxonomic units (OTU) at 97% similarity threshold. Taxonomic ranks were assigned with the Ribosomal Database Project (RDP) Naïve Bayesian Classifier v.2.2. α- and β-diversity were analyzed based on OTUs and their taxonomic ranks.

### NGS data analysis: RNA-seq sorted cell, colon crypts, SI-tissue, and SI-organoids

RNA-seq reads were adaptor trimmed with Trim Galore (v0.4.4, v0.4.2 for organoid and colon crypt data, v0.4.1 for SI-whole tissue data). Reads were mapped to the mouse reference genome GRCm38/mm10 with HiSat2 (v2.1.0, no soft clipping, v2.0.3 for organoid data, v2.0.1 for SI-whole tissue and colon crypt data). Uniquely mapped RNA-seq data was analyzed with SeqMonk (v1.43.0, v1.42.0 for organoid, SI-whole tissue, and colon crypt data). Read counts were quantified over exons of merged transcripts using the SeqMonk RNA-seq quantitation pipeline. DEG were identified based on the raw read count quantitation over merged transcript isoforms with the multiple testing corrected DESeq2 algorithm in SeqMonk. Fold changes were quantified after RPKM (Reads Per Kilobase of transcript per Million mapped reads) normalization over merged transcript isoforms in SeqMonk.

### NGS data analysis: RNA-seq DSS in colon

RNA-seq reads were mapped to the mouse reference genome GRCm38/mm10 with HiSat2 (version 2.1.0). Uniquely mapped RNA-seq data was analyzed with SeqMonk version 1.42.0. Read counts were quantified over exons of merged transcripts using the SeqMonk RNA-seq quantitation pipeline. As rRNA contamination was detected in 3 samples, rRNA-annotated reads were filtered (using an rRNA annotation track, submitted with dataset to GEO). DEG were identified based on the raw read count quantitation over merged transcript isoforms with the multiple testing corrected DESeq2 algorithm in SeqMonk. Fold changes were quantified after RPKM normalization over merged transcript isoforms in SeqMonk. Gene expression clusters were identified using SeqMonk per-probe normalized hierarchical clustering. GO enrichment analysis of DEG was performed with g:profiler against the indicated background lists.

## Supplementary information

**Additional file 1.** Supplementary Figures S1-S11.

**Additional file 2: Table S1.** Full statistical analysis data and annotation for the Fig. 3d, 4c, 5b, 6a,b,d, 7a and Additional file 1: Figures S1b-d, S3b,d-f, S4b,c, S6c-f.

**Additional file 3: Table S2.** Differentially expressed genes on Smarcad1-KO in ISC, TA, AE and small intestine derived organoids. Gene lists and their overlaps as shown in Additional file 1: Fig. S2a. Table contents: Gene name, chromosomal position, DESeq2 FDR, gene ID, gene description, average expression levels (*n* = 3, log2 normalized to total reads) in the corresponding RNA-seq dataset. Separate tab comparing the 4 lists (ISC, TA, AE, organoids). Separate tabs listing all genes expressed in small intestinal organoids and colon crypts (sorted cell and whole crypt datasets combined).

**Additional file 4: Table S3.** Differentially expressed genes on *Smarcad1*-KO in whole colon crypts and flow cytometry-sorted colon epithelium. Gene lists and their overlaps as shown in Additional file 1: Fig. S2b. Table contents: Gene name, chromosomal position, DESeq2 FDR, gene ID, gene description, average/by sample expression levels (*n* = 3, log2 normalized to total reads) in the corresponding RNA-seq dataset. Separate Tab comparing the 2 lists (crypts, sorted epithelial cells).

**Additional file 5: Table S4.** Genes identified with the DESeq2 test (cut-off FDR < 0.05) on comparison of RNA-seq datasets from WT and *Smarcad1*-KO (*Villin-cre* mediated) small intestinal whole tissue samples. Table contents: Gene name, chromosomal position, DESeq2 FDR and expression levels (*n* = 4 log2, RPKM) in WT/KO datasets by sample and as WT/KO average.

**Additional file 6: Table S5.** Differential H3K9me3 MACS-peaks identified with the EdgeR test (cut-off FDR < 0.00001) on comparison of ChIP-seq datasets from WT and *Smarcad1*-KO (*Villin-cre* mediated) small intestinal whole crypt isolates. Table contents: MACS-peak chromosomal position, closest gene (5 kbp cut-off) with distance and annotation, EdgeR FDR (Benjamini-Hochberg corrected) and quantitation (*n* = 3, log2, lower limit 1.0, transformed by matching distributions) in WT/KO datasets by sample, log2 fold-changes in WT/KO.

**Additional file 7: Table S6.** Differential H3K9me2 MACS-peaks (300 bp fragment size) identified with the EdgeR test (cut-off FDR < 0.00001) on comparison of ChIP-seq datasets from WT and *Smarcad1*-KO (*Villin-cre* mediated) small intestinal whole crypt isolates. Table contents: MACS-peak chromosomal position, closest gene (5 kbp cut-off) with distance and annotation, EdgeR FDR (Benjamini-Hochberg corrected) and quantitation (n = 3, log2, lower limit 1.0, transformed by matching distributions) in WT/KO datasets by sample.

**Additional file 8: Table S7.** Differential H3K9me3 MACS-peaks identified with the EdgeR test (cut-off FDR < 0.00001) on comparison of ChIP-seq datasets from WT and *Smarcad1*-KO (*Villin-cre* mediated) colon whole crypt isolates. Table contents: MACS-peak chromosomal position, closest gene (5 kbp cut-off) with distance and annotation, EdgeR FDR (Benjamini-Hochberg corrected) and quantitation (n = 3, log2, lower limit 1.0, transformed by matching distributions) in WT/KO datasets by sample.

**Additional file 9: Table S8.** Differential chromatin accessibility MACS-peaks (300 bp fragment size) identified with the EdgeR test (cut-off FDR < 0.00001) on comparison of ATAC-seq datasets from WT and *Smarcad1*-KO (*Villin-cre* mediated) small intestinal whole crypt isolates. Table contents: MACS-peak chromosomal position, closest gene (5 kbp cut-off) with distance and annotation, EdgeR FDR (Benjamini-Hochberg corrected) and quantitation (n = 3, log2, read counts normalized to largest data store) in WT/KO datasets by sample.

**Additional file 10: Table S9.** Supplementary phenotype data for the DSS-induced colitis experiments (Fig. 6a, b and Additional file 1: Fig. S6a, b). DAI (disease activity index) scores were determined for each experiment separately, based on weight loss and a selection of fecal blood, stool consistency and overall animal appearance scores. Detailed scoring tables described in the according tab. Colon length after DSS-induction was determined in experiment 2 (Fig. 6b and Additional file 1: Fig. S6b).

**Additional file 11: Table S10.** Differentially expressed genes on *Smarcad1*-KO in control, microbiome enriched whole colon tissue samples. Gene lists as shown in Additional file 1: Fig. S9a. Table contents: Gene name, chromosomal position, DESeq2 FDR (cut-off 0.05), gene ID, gene description, average expression levels (log2 normalized to total reads) in WT/KO control (n = 3) and colitis (*n* = 5) data sets.

**Additional file 12: Table S11.** Differentially expressed genes on *Smarcad1*-KO in microbiome enriched, DSS-induced colitis, whole colon tissue samples. Gene lists as shown in Additional file 1: Fig. S9b. Table contents: Gene name, chromosomal position, DESeq2 FDR (cut-off 0.05), gene ID, gene description, average expression levels (log2 normalized to total reads) in WT/KO control (n = 3) and colitis (n = 5) data sets.

**Additional file 13: Table S12.** Genes identified with the DESeq2 test (cut-off FDR < 0.05) on comparison of RNA-seq datasets from control and DSS-induced samples. WT samples after microbiome enrichment. Table contents: Gene name, chromosomal position, DESeq2 FDR and expression change on colitis induction (UP/DOWN).

**Additional file 14: Table S13.** Subset of gene list in Additional file 13: Table S12, indicated as cluster A in Fig. 8a. Genes were identified by hierarchical clustering in SeqMonk. Table contents: Gene name, chromosomal position.

**Additional file 15: Table S14.** Subset of gene list in Additional file 13: Table S12, indicated as clusters 1–4 in Fig. 8b. Genes were identified by hierarchical clustering in Seqmonk. Table contents: Gene name, selected annotations, cluster membership.

**Additional file 16: Table S15.** List of genes with colonic expression (whole tissue, see Fig. 8), defined as detectable expression in either all WT/KO ctrl or all WT/KO colitis samples. Table contents: Gene name, chromosomal position, normalized linear read counts by sample.

**Additional file 17: Table S16.** List of enriched gene ontology terms of clusters 2 (Additional file 15: Table S14) versus all genes expressed in the colon (Additional file 16: Table S15) with a statistical cutoff *p* < 0.1. Table contents: full g:profiler results and annotation, permanent link to the analysis with updated annotation database access.

**Additional file 18: Table S17.** List of enriched gene ontology terms of clusters 2 (Additional file 15: Table S14) versus all genes significantly upregulated in WT upon colitis (marked as UP in Additional file 13: Table S12) with a statistical cutoff *p* < 0.2. Table contents: full g:profiler results and annotation, permanent link to the analysis with updated annotation database access.

**Additional file 19: Table S18.** List of enriched gene ontology terms of cluster A (Additional file 14: Table S13) versus all genes significantly upregulated in WT upon colitis (Additional file 13: marked as UP in Table S12) with a statistical cutoff *p* < 0.05. Table contents: full g:profiler results and annotation, permanent link to the analysis with updated annotation database access.

**Additional file 20: Table S19.** List of enriched gene ontology terms of clusters 3/4 (Table S14) versus all genes expressed in the colon (Additional file 16: Table S15) with a statistical cutoff p < 0.1. Table contents: full g:profiler results and annotation, permanent link to the analysis with updated annotation database access.

**Additional file 21: Table S20.** List of enriched gene ontology terms of *WT* colon genes upregulated in colitis (Additional file 13: Table S12, marked UP) versus all genes expressed in the colon (Additional file 16: Table S15) with a statistical cutoff p < 0.05. Table contents: full g:profiler results and annotation, permanent link to the analysis with updated annotation database access.

**Additional file 22: Table S21.** Principle Component Analysis based on OTU abundance in stool samples. Full list of 42 principal component values by sample. PC1 and PC2 shown in Additional file 1: Fig. S8a.

**Additional file 23: Table S22.** All detected OTUs annotated with counts per sample. Sublists marked in Additional file 1: Fig. S8b are marked as follows: red: OTUs not transferred from donor to recipients. Green: Candidate OTUs for enhanced colitis response and Smarcad1-mediated susceptibility. Table contents: OTU ID, detection counts per sample, digital indicators of presence in initial, donor and enriched microbiota, indicators of transferred (green) and un-transferred (red) OTUs, taxonomic information.

**Additional file 24: Table S23.** EdU assay example files and Excel-script for calculation of EdU-signal to crypt base distance.

**Additional file 25: Protocol S1.** ImageJ script and Excel annotation for the distance quantification of EdU signals on fluorescent images of the intestine as shown in Additional file 1: Fig. S1e-f. Related example files and Excel script in Additional file 24 Table S23.

**Additional file 26.** Review history.

## Data Availability

Next generation sequencing data are available in the NCBI GEO, under accession number GSE127556 [91]. The authors declare that all other data supporting the findings of this study are within the manuscript and its supplementary files.
