# Supplementary material for: Smarcad1 mediates microbiota-induced inflammation in mouse and coordinates gene expression in the intestinal epithelium
Source: Genome Biol. 2020 Mar 11;21:64. doi: 10.1186/s13059-020-01976-7 (PMC7065452; doi:10.1186/s13059-020-01976-7)
Supplement: Supplementary file 1 — Additional file 1. Supplementary Figures S1-S11. [file 13059_2020_1976_MOESM1_ESM.pdf]

Smarcad1 mediates microbiota-induced inflammation in mouse and coordinates gene expression in the intestinal epithelium

Juri Kazakevych[1], Jérémy Denizot[1,2], Anke Liebert[1,3], Mariana Portovedo[4], Mia Mosavie[5], Payal Jain[1], Claudia Stellato[1], Claire Fraser[1], Renan Oliveira Corrêa[4], Marina Célestine[1], Raphaël Mattiuz[1], Hanneke Okkenhaug[6], J. Ross Miller[1], Marco Aurélio Ramirez Vinolo[4], Marc Veldhoen[7,8] and Patrick Varga-Weisz[1,5*]

[1]Nuclear Dynamics, Babraham Institute, Cambridge, CB22 3AT, UK
[2]Current address: Université Clermont Auvergne, Inserm U1071, INRA USC2018, M2iSH, F-63000 Clermont–Ferrand, France
[3]Current address: The Francis Crick Institute, London, NW1 1AT, UK
[4]Lab. of Immunoinflammation, Institute of Biology, UNICAMP, Campinas, 13083-862, Brazil
[5]School of Biological Sciences, University of Essex, Colchester, CO4 3SQ, UK
[6]Imaging Facility, Babraham Institute, Cambridge, CB22 3AT, UK
[7]Lymphocyte Signalling and Development, Babraham Institute, Cambridge, CB22 3AT, UK
[8]Current address: Instituto de Medicina Molecular | João Lobo Antunes, Faculdade de Medicina da Universidade de Lisboa, Lisbon, 1649-028, Portugal
*correspondence: patrick.varga-weisz@essex.ac.uk

Additional file 1:

Figures S1-S11 and corresponding legends

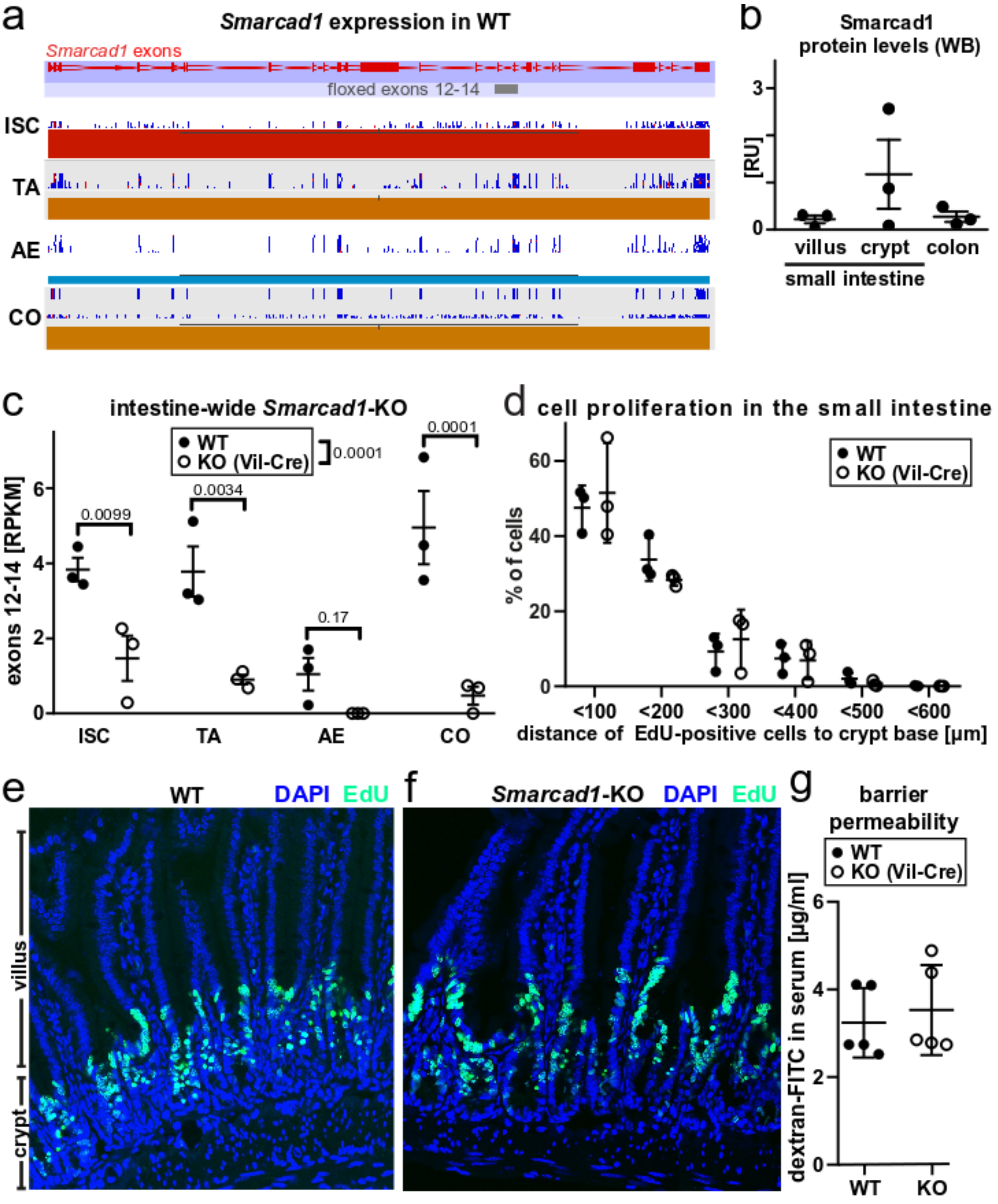

Figure S1: Additional validation of *Smarcad1*-KO. a *Smarcad1*-expression in WT SI stem cells (ISC), transit amplifying cells (TA), adult enterocytes (AE) and colon epithelial cells (CO) by RNA-seq (n=3) as aligned reads and logarithmic quantitation (blue-red gradient). Floxed exons 12-14 targeted by intestinal epithelium specific *Vil-cre* mediated KO are indicated in grey. **b** Quantitation of Western blots (n=3, example shown in Fig. 1f) on WT

samples in SI villus/crypt and colon normalized to H3 loading control (>15 kDa band). Anterior, central and posterior fractions of SI villus/crypt samples were treated as technical replicates. SEM indicated by error bars. Statistical test by 2-way ANOVA with Holm-Sidak's multiple comparison test shown in Additional file 2: Table S1. **c** RNA-seq quantitation of expression of the floxed exons 12-14 (1873 bp) in ISC, TA, AE and CO of WT and KO animals. Linear read quantitation RPKM-normalized with SEM indicated by error bars. Statistical test by 2-way ANOVA with Holm-Sidak's multiple comparison test. **d** Quantitation of proliferative cell distance to crypt bottom. No significant differences between WT and KO (2-way ANOVA, Holm-Sidak multiple testing correction, P>0.8, SD indicated by error bars, n=3). **b-d** Full statistical results are listed in Additional file 2: Table S1. **e, f** Intestinal localization of proliferative cells by EdU-assay with AlexaFuor488 (green) and nuclear counterstaining with DAPI (blue). Scale bars: 20 µm. Representative WT (**e**) and Smarcad1-KO (**f**) samples. **g** Quantitation of FITC-dextran serum levels indicate KO-unaffected barrier function of the intestinal epithelium. 10 mg FITC-dextran were applied per animal for 5 h, serum sampled in heparin tubes and fluorescence (emission 488 nm, excitation 522 nm) measured relative to a standard curve.

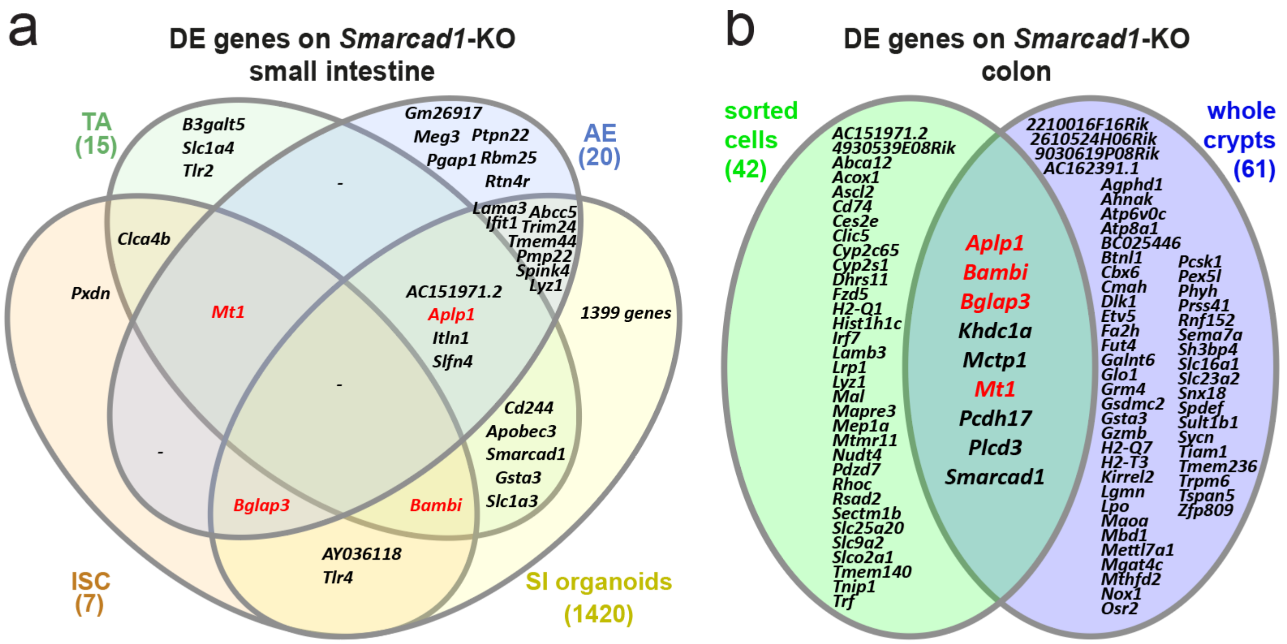

Figure S2: Hallmark genes on intestinal *Smarcad1*-KO deletion. Venn diagrams of gene lists identified by DESeq2. **a** RNA-seq data sets from small intestinal cell types (AE: adult enterocytes, TA: transit amplifying cells, ISC: intestinal stem cells, n=3, DESeq2 FDR cut-off <0.1) and small intestinal organoids (n=3, DESeq2 FDR cut-off <0.05). 23 genes appear in at least 2 DEG (differentially expressed genes) lists. See Additional file 3: Table S2 for full gene lists and statistics. **b** RNA-seq data sets from flow-sorted colon epithelial cells and whole colon crypts (n=3, DESeq2 FDR cut-off <0.05). See Additional file 4: Table S3 for full gene lists and statistics. **a, b** Total gene number per list is shown in brackets. Genes appearing in the DE list overlaps in both, the small intestine and colon, are marked red.

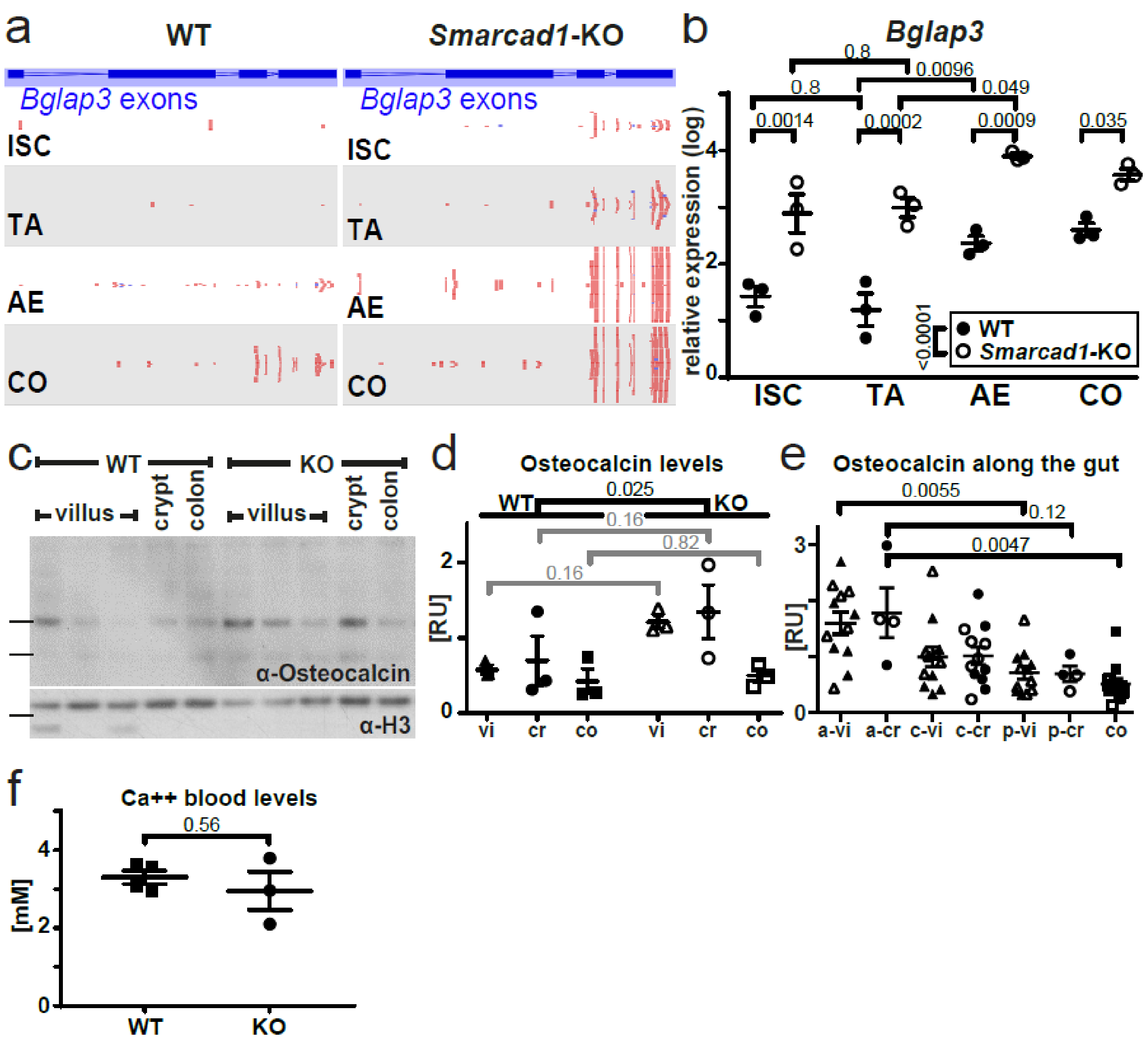

Figure S3: Osteocalcin in the intestinal tract of WT and *Smarcad1*-KO mice. a *Bglap3*-expression in WT and *Vil-cre* mediated *Smarcad1*-KO SI stem cells (ISC), transit amplifying cells (TA), adult enterocytes (AE) and colon epithelial cells (CO) as representative RNA-seq tracks. **b** RNA-seq quantitation of *Bglap3* in ISC, TA, AE and CO of WT and *Smarcad1*-KO animals (n=3). **b, d, e:** SEM indicated by error bars. Statistical test by 2-way ANOVA with Holm-Sidak's multiple comparison test, P-values indicated for selected comparisons. Full statistical results are listed in Additional file 2: Table S1. **c** Representative Western blot of Osteocalcin and H3 loading normalization in WT (left) and KO (right) samples. Villus SI samples are shown as anterior, central and posterior fractions of the SI. Relative molecular weights 25 kDa, 20 kDa and 15 kDa are indicated on the left (top to bottom). **d** Quantitation

of Western blots (n=3) on WT and *Smarcad1*-KO samples in SI villus (vi), SI crypt (cr) and colon (co) normalized to H3 loading control (>15 kDa band). Anterior-posterior fractions of villus samples were treated as technical replicates. **e** Anterior-posterior gradient of Osteocalcin. Quantitation of Western blots on WT and *Smarcad1*-KO samples in SI villus (vi), SI crypt (cr) and colon (co) (n=2-6) normalized to H3 loading control and per-sample average. The anterior-posterior decrease of Osteocalcin levels is independent of *Smarcad1*-KO (P=0.70). **f** Ca^{++} blood levels in WT and *Smarcad1*-KO animals (n=3-4, Unpaired t test with Welch's correction, SEM indicated by error bars, statistics are listed in Additional file 2: Table S1). 200 µl blood from 12-17 weeks old mice was taken from the heart and mixed with 10 U heparin. The Colorimetric Calcium Assay Kit (Abcam 102505) was used according to manufacturers specifications.

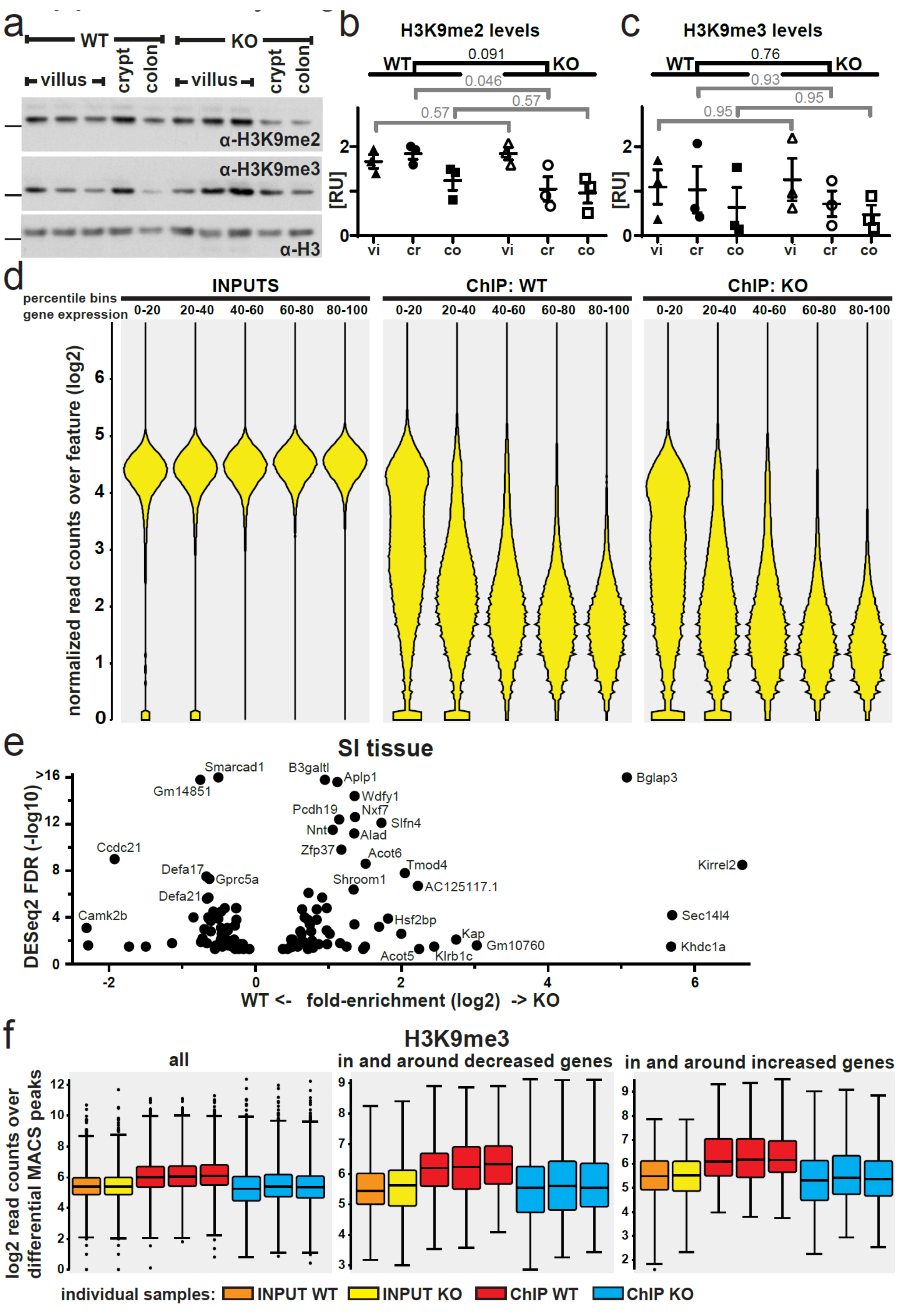

a
WT
KO
villus
crypt
colon
α-H3K9me2
α-H3K9me3
α-H3
b
H3K9me2 levels
0.091
0.046
0.57
0.57
[RU]
vi
cr
co
c
H3K9me3 levels
0.76
0.93
0.95
0.95
d
percentile bins gene expression
INPUTS
ChIP: WT
ChIP: KO
0-20
20-40
40-60
60-80
80-100
normalized read counts over feature (log2)
e
SI tissue
DESeq2 FDR (-log10)
WT <- fold-enrichment (log2) -> KO
Smarcad1
Gm14851
B3galtl
Aplp1
Bglap3
Wdfy1
Pcdh19
Nxf7
Slfn4
Nnt
Alad
Ccdc21
Zfp37
Acot6
Kirrel2
Defa17
Gprc5a
Tmod4
Shroom1
AC125117.1
Defa21
Camk2b
Hsf2bp
Sec14l4
Kap
Gm10760
Khdc1a
Acot5
Klrb1c
f
H3K9me3
all
in and around decreased genes
in and around increased genes
log2 read counts over differential MACS peaks
individual samples:
INPUT WT
INPUT KO
ChIP WT
ChIP KO

Figure S4: H3K9me2/3 in the small intestine epithelium of WT and *Smarcad1*-KO mice. **a** Representative Western blots of H3K9me2, H3K9me3 and H3 loading normalization in WT (left) and KO (right) samples. Villus SI samples are shown as anterior, central and posterior fractions along the SI. Relative molecular weight 15 kDa is indicated on the left. **b, c** Quantitation of H3K9me2 (**b**) and H3K9me3 (**c**) Western blots (n=3) on WT and *Smarcad1*-KO samples in SI villus (vi), SI crypt (cr) and colon (co) normalized to H3 loading control. Anterior-posterior fractions of villus samples were treated as technical replicates. SEM indicated by error bars. Statistical test by 2-way ANOVA with Holm-Sidak's multiple comparison test, P-values indicated. Full statistical results are listed in Additional file 2: Table S1. **d** H3K9me3 is enriched over promoters of silent genes. The average H3K9me3 signals of all WT and KO libraries are shown as ChIP-seq read counts +/- 1000 bp around gene start sites. These were binned according to percentile transcript abundance using whole small intestine tissue RNAseq data (see e), corrected for gene length. The y-axis was capped at (log2) 7. **e** Significantly up/down-regulated genes in small intestinal tissue RNA-seq (DESeq2 test with cutoff FDR<0.05, n=4). X-axis: *Smarcad1*-KO/WT log(2) fold-enrichment of reads per transcript. Y-axis: -log(10) DESeq2 FDR. High FDR values capped at indicated max. Y-values for visualization. 0-expression values were set to 0.1 to allow log-plotting of fold-changes. For additional data and annotations see Additional file 5: Table S4. **f** *Smarcad1*-KO is generally linked to loss of H3K9me3 around genes in the intestinal epithelium. Differential (EdgeR p<0.05) MACS peaks for H3K9me3 were identified within 5 kbp up/down-stream of the indicated genes and read counts quantified: all genes, and only genes whose expression is down-regulated or up-regulated on *Smarcad1*-KO. Shown are the results by biological replicate. All samples, but not input, were normalized with 'match distribution' function of SeqMonk. The DEG list was from the small intestinal organoid data set (see Fig. 2f, Additional file 3: Table S2).

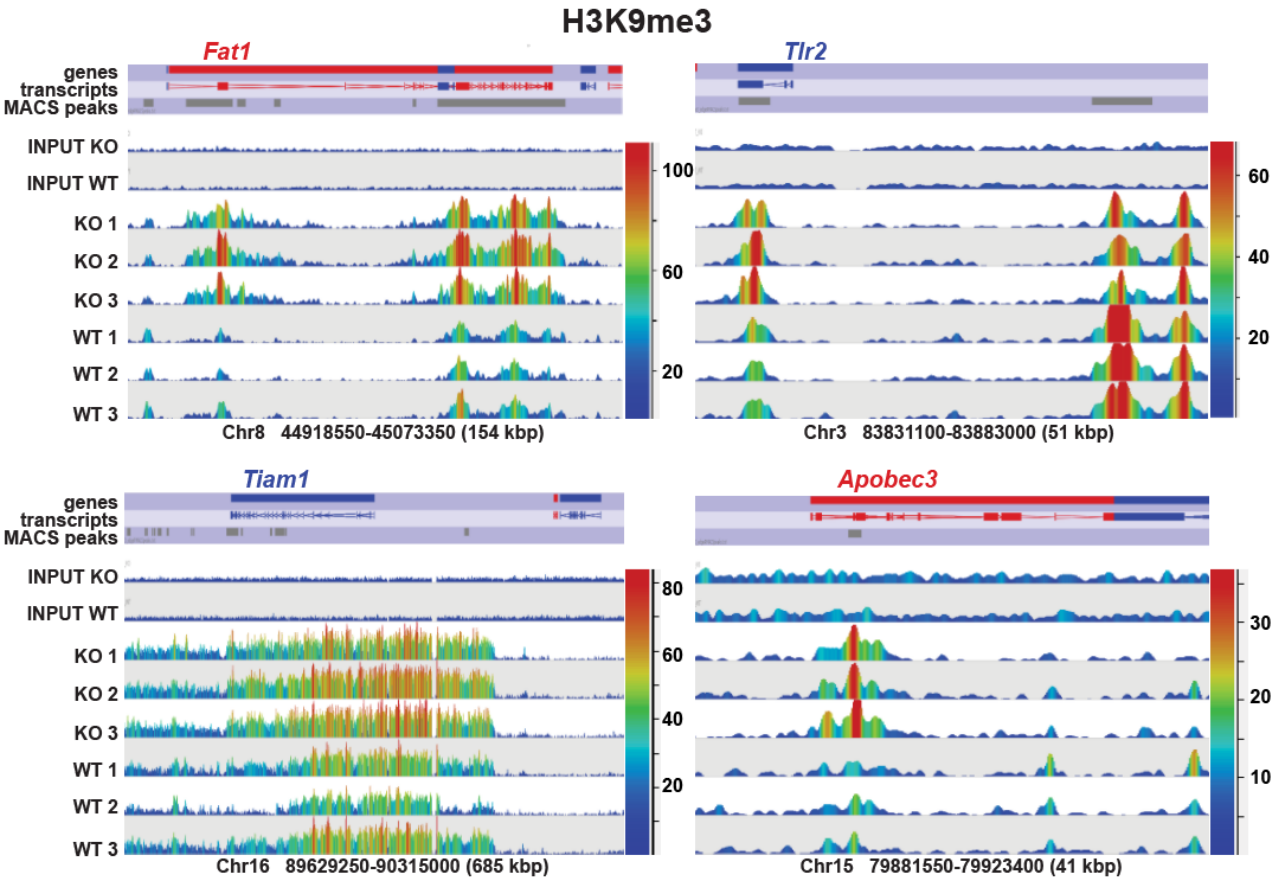

Figure S5: Additional examples of small intestinal H3K9me3 changes over gene bodies, where H3K9me3 increases upon *Smarcad1*-KO. The top annotation track indicates genes, the second track transcripts and the third MACS peaks. Read counts are shown in linear scale, color coded. Chromosomal positions and window sizes in kbp are indicated.

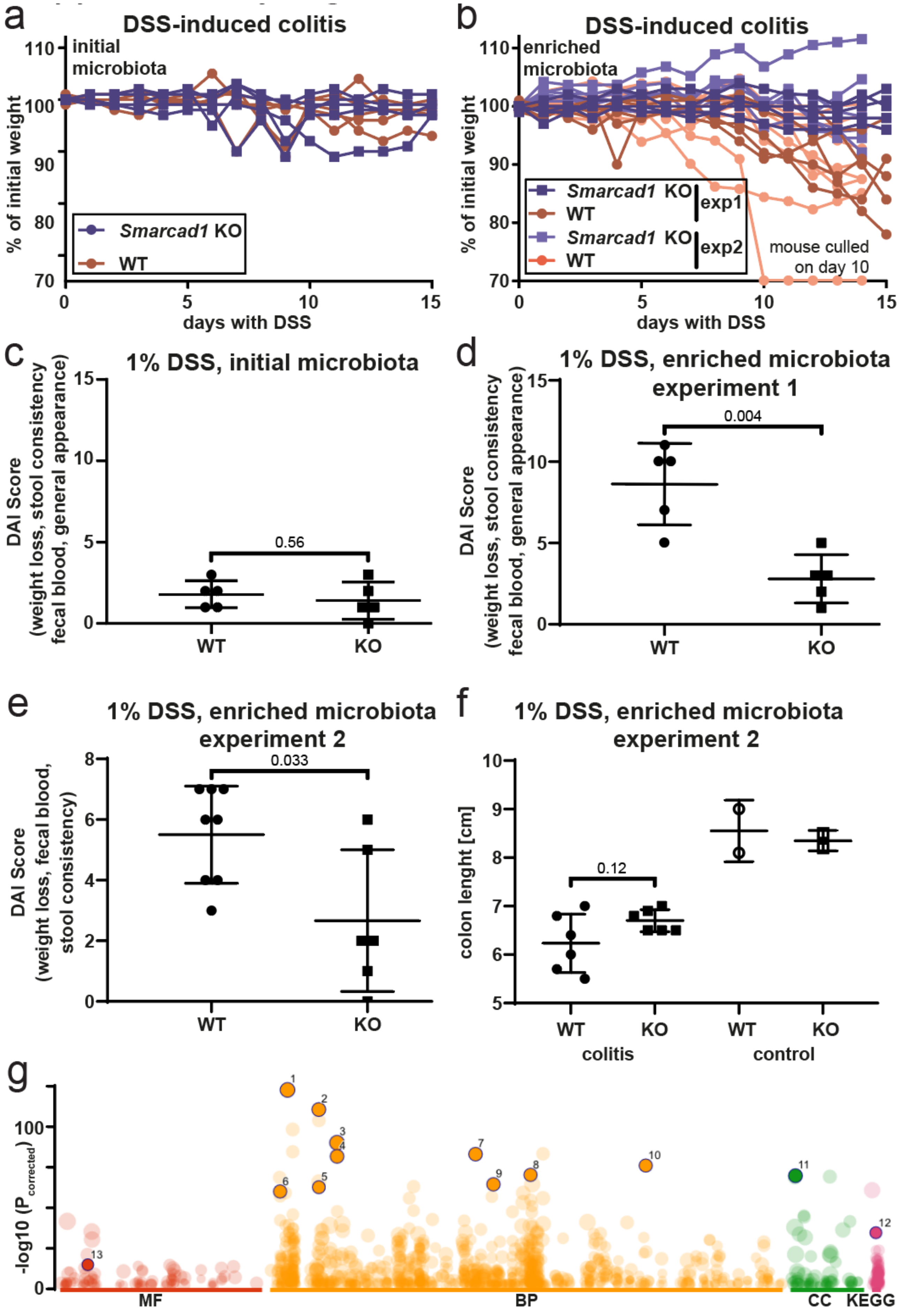
a
DSS-induced colitis
initial microbiota
% of initial weight
days with DSS
Smarcad1 KO
WT
b
DSS-induced colitis
enriched microbiota
% of initial weight
days with DSS
Smarcad1 KO
WT
exp1
Smarcad1 KO
WT
exp2
mouse culled on day 10
c
1% DSS, initial microbiota
DAI Score (weight loss, stool consistency fecal blood, general appearance)
0.56
WT
KO
d
1% DSS, enriched microbiota experiment 1
DAI Score (weight loss, stool consistency fecal blood, general appearance)
0.004
WT
KO
e
1% DSS, enriched microbiota experiment 2
DAI Score (weight loss, fecal blood, stool consistency)
0.033
WT
KO
f
1% DSS, enriched microbiota experiment 2
colon lenght [cm]
0.12
WT
KO
WT
KO
colitis
control
g
-log10 (P corrected)
MF
BP
CC
KEGG

Figure S6: Smarcad1-dependent colitis susceptibility. a-f Detailed results of experiments shown in **Figure 6.** 1% DSS-induced colitis phenotype in WT and *Smarcad1*-KO animals. Animal weights are normalized to initial weight on day 0 (**b-2**) or on the average of 3 consecutive measurements on days -2, -1, 0 (**a**, **b-1**). **a**, **c** Initial (non-enriched) microbiota. Experiment terminated after 15 days (n=5 for WT/ KO). **b**, **d-f** 1% DSS-induced colitis phenotype in WT and *Smarcad1*-KO animals with enriched microbiota, 2 independent experiments shown. Experiment 1 terminated after 15 days (n=5 for WT/KO), Experiment 2 after 14 days with one mouse culled after 10 days due to extensive weight loss (n=8 for WT, n=6 for KO). **c-f:** SD indicated by error bars. Indicated P-values determined by unpaired t-test with Welch's correction. **c-e** End-of-experiment colitis DAI (disease activity index) scored separately by experiment. **f** Colon-shortening on DSS-induced colitis. Full phenotyping data and scoring tables are listed in Additional file 10: Table S9, full statistical results are listed in Additional file 2: Table S1. **g** DE genes on DSS-induced colitis confirm inflammatory response. GO-enrichment analysis of genes upregulated on colitis in WT (Additional file 13: Table S12, marked UP) versus genes expressed in the colon (Additional file 16: Table S15). BP: biological process, CC: cellular component, MF: molecular function, KEGG: KEGG biological pathways. Significance threshold corrected P=0.05. Selected enriched terms are indicated: immune system process (1), defense response (2), resonse to external stimulus (3), response to biotic stimulus (4), inflammatory response (5), cytokine production (6), response to external biotic stimulus (7), regulation of immune response (8), leukocyte activation (9), defense response to other organism (10), extracellular space (11), cytokine-cytokine receptor interaction (12) and cytokine receptor binding (13). Full gene ontology enrichment analysis is listed in Additional file 21: Table S20**.**

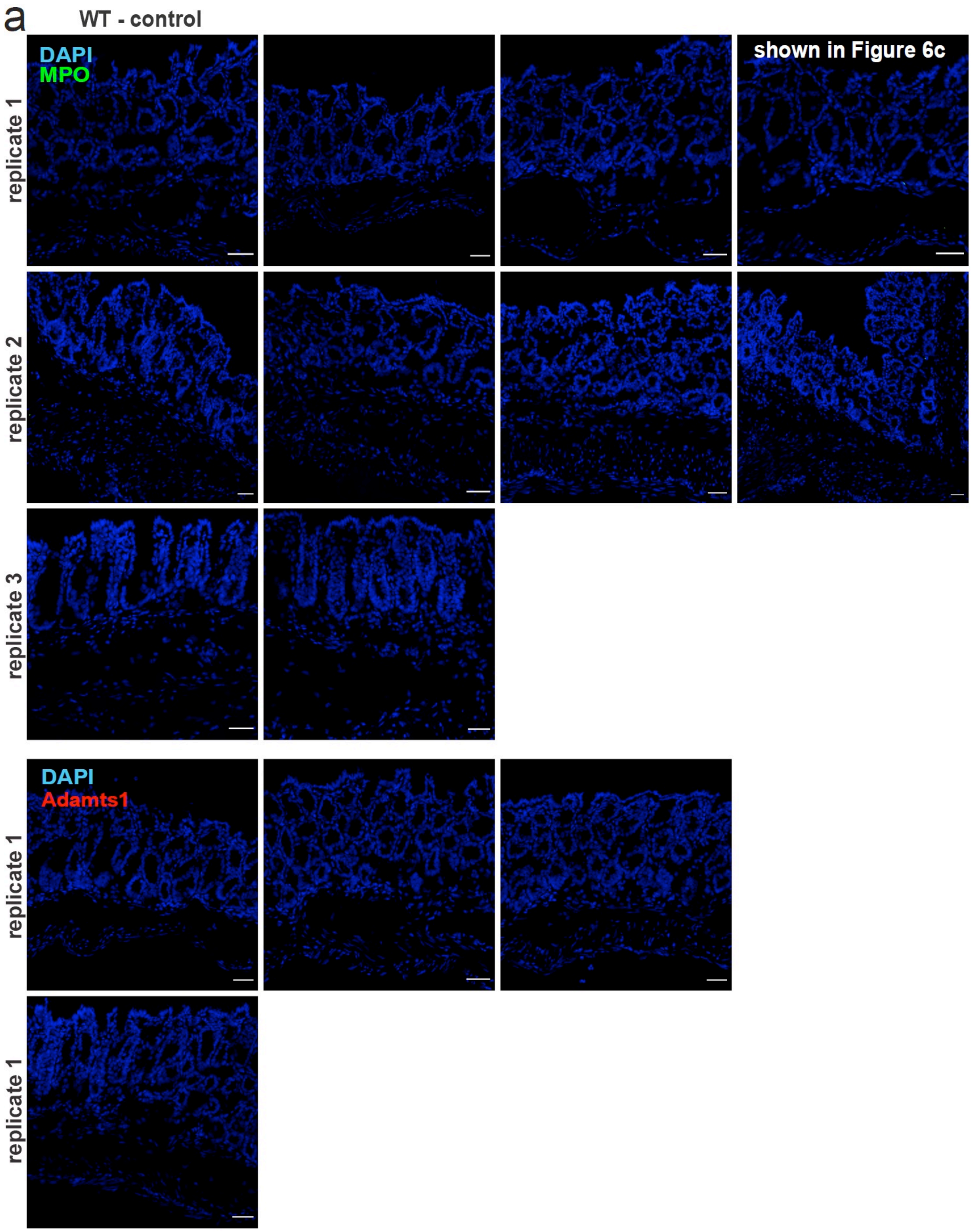
a
WT - control
DAPI
MPO
shown in Figure 6c
replicate 1
replicate 2
replicate 3
DAPI
Adamts1
replicate 1
replicate 1

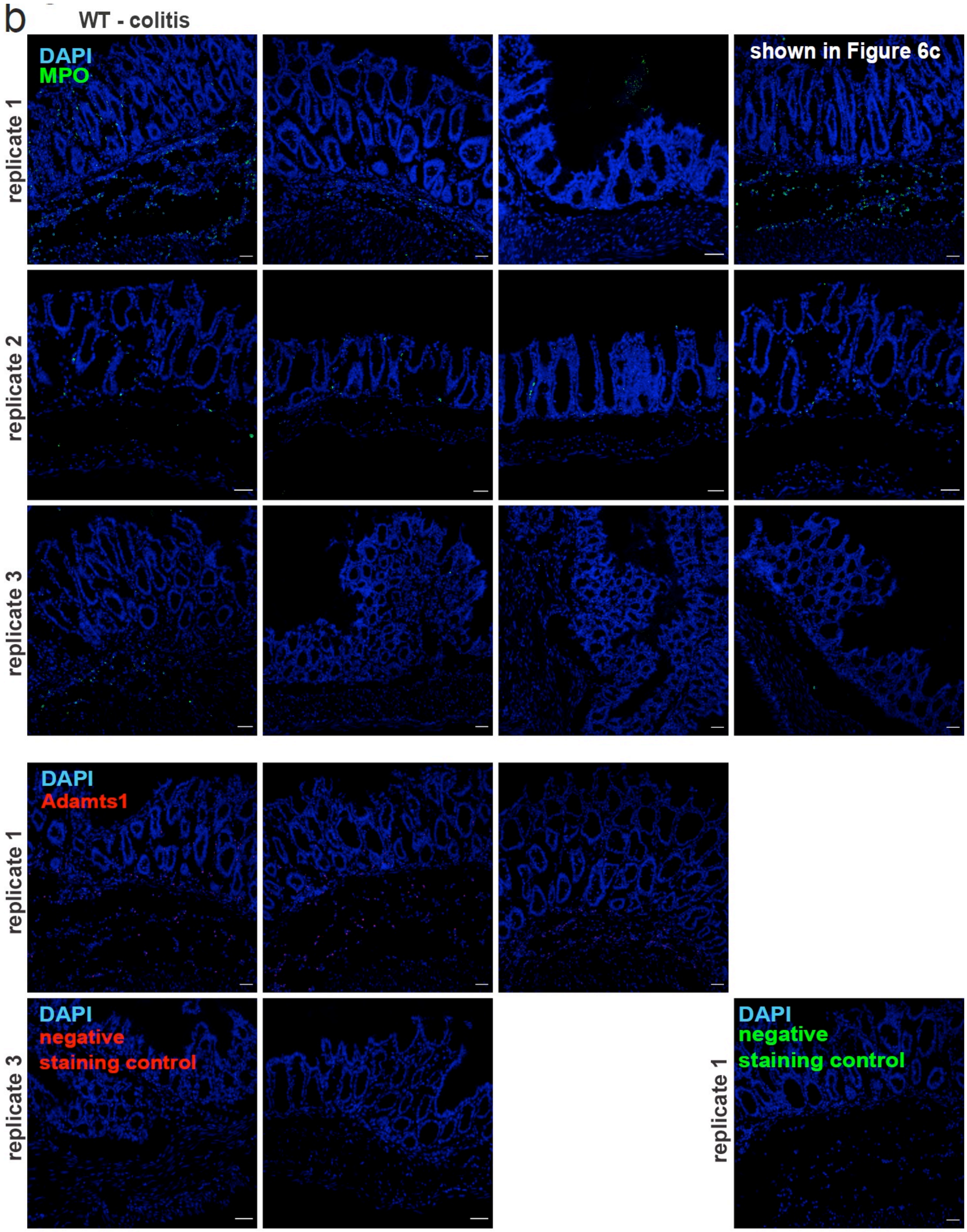
b
WT - colitis
DAPI
MPO
shown in Figure 6c
replicate 1
replicate 2
replicate 3
DAPI
Adamts1
replicate 1
DAPI
negative
staining control
replicate 3
DAPI
negative
staining control
replicate 1

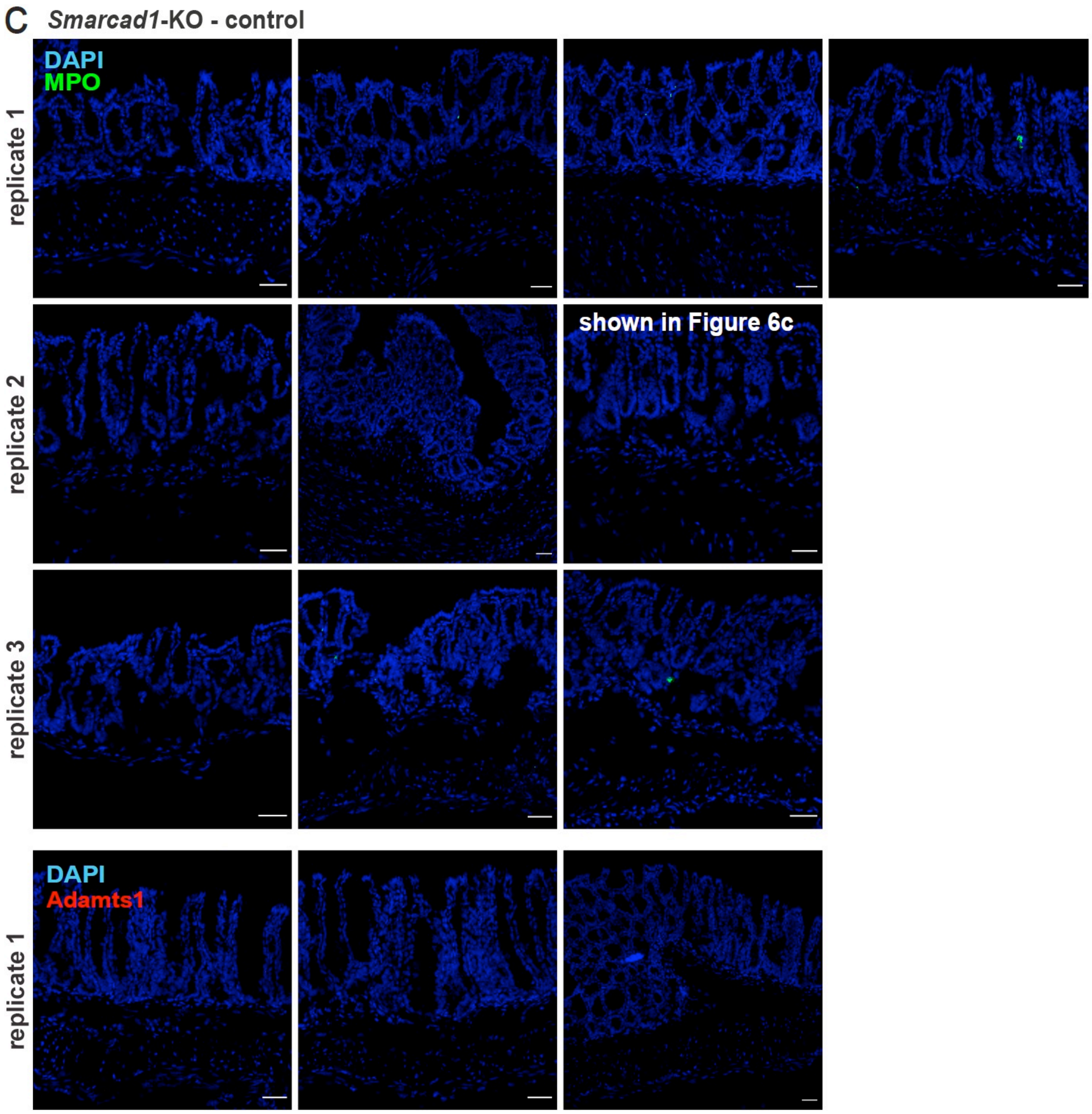
C
Smarcad1-KO - control
DAPI
MPO
replicate 1
replicate 2
shown in Figure 6c
replicate 3
DAPI
Adamts1
replicate 1

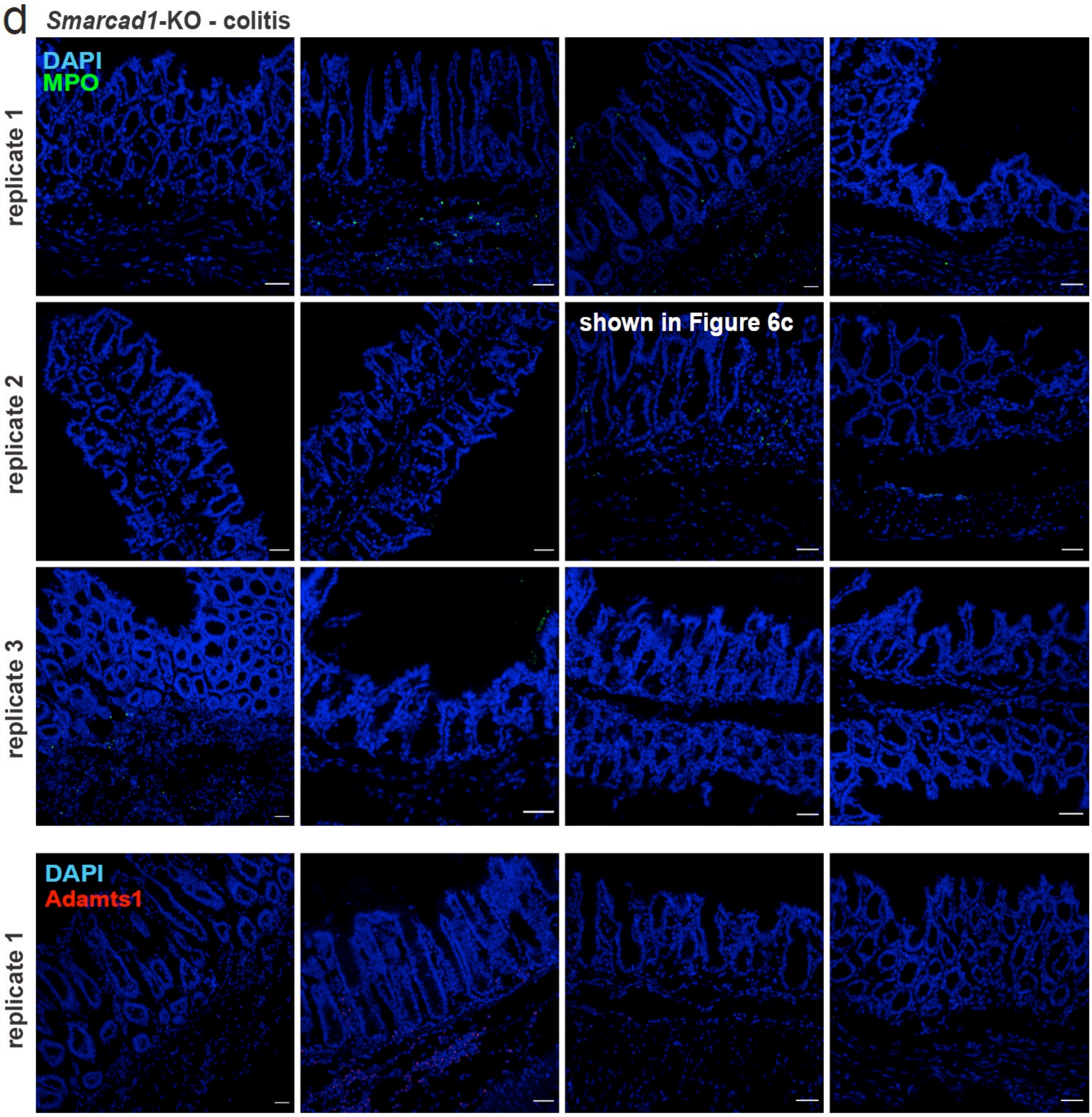
d
Smarcad1-KO - colitis
DAPI
MPO
replicate 1
replicate 2
shown in Figure 6c
replicate 3
DAPI
Adamts1
replicate 1

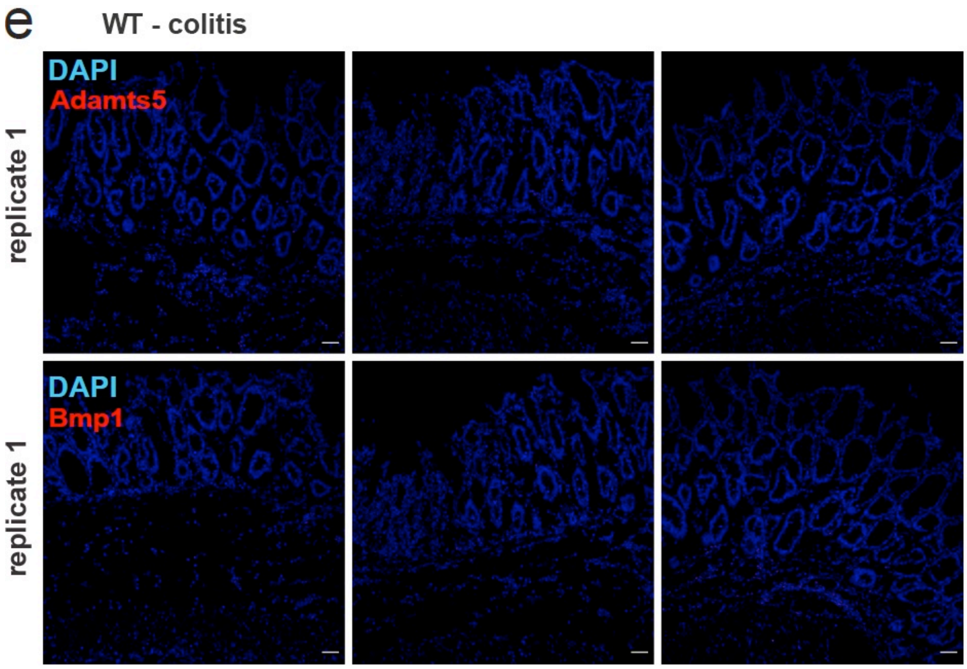

Figure S7: Immune cell recruitment on colitis induction by 1% DSS. a-e Intestinal colon epithelium localization of MPO-positive cells (green, neutrophil/lymphocyte-marker) (**a-d**) and localization of the Adamts1 (**a-d**) /Adamts5 (**e**) /Bmp1 (**e**) proteases (red) by IF staining and nuclear counterstaining with DAPI (blue) shown as maximum intensity projections. 14-day colitis induction with enriched microbiota: WT control (**a**), WT colitis (**b,e**), KO control (**c**) and KO colitis (**d**). No primary antibody control on WT control and colitis samples are indicated. Scale bars: 40 µm. WT: wild type. KO: Vil-cre mediated tissue specific knockout of Smarcad1 (KO). Biological replicates indicated with all technical replicates shown. This image set is partially shown in Fig. 6c and quantified in Fig. 6d.

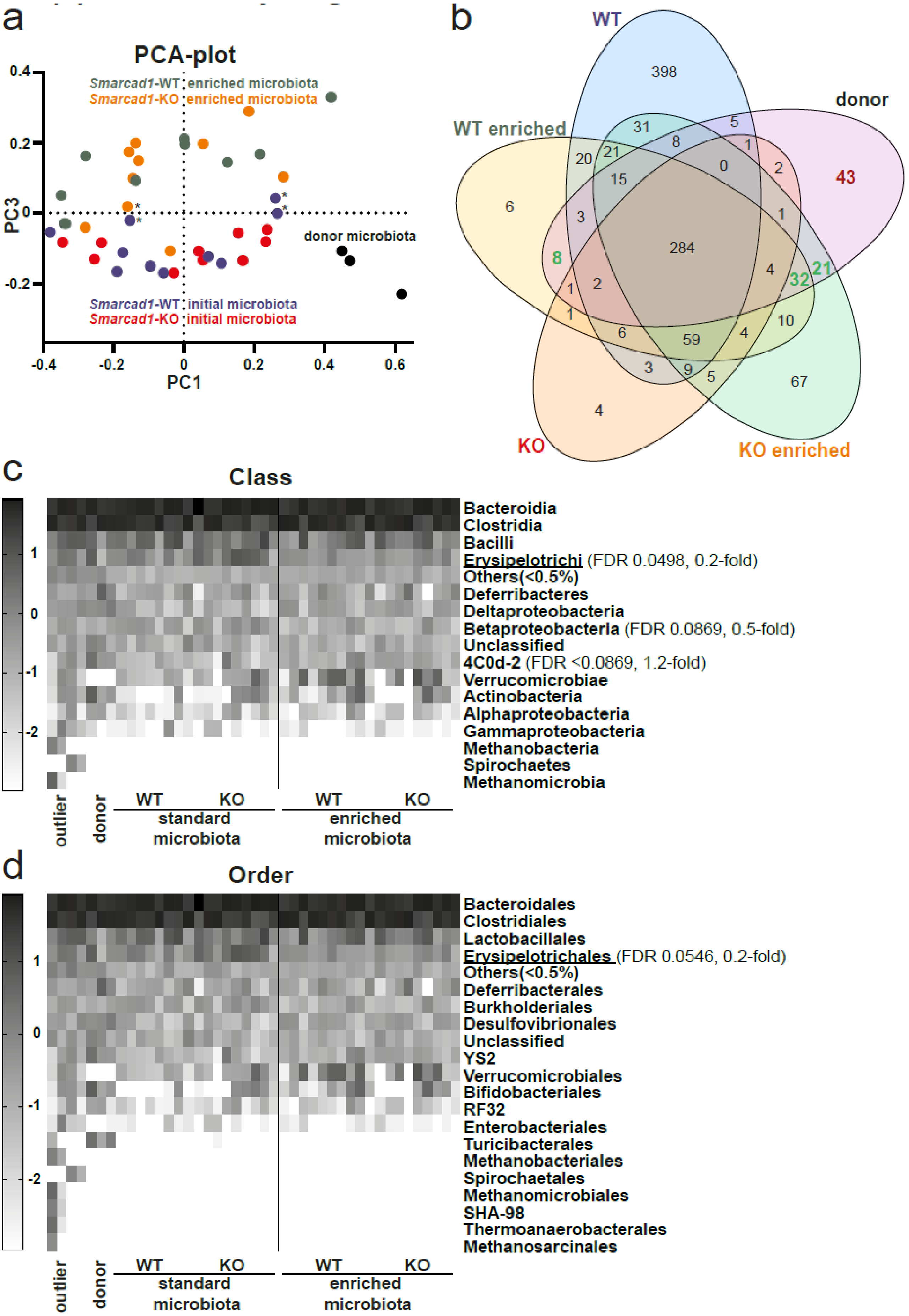
a
PCA-plot
Smarcad1-WT enriched microbiota
Smarcad1-KO enriched microbiota
donor microbiota
Smarcad1-WT initial microbiota
Smarcad1-KO initial microbiota
PC3
PC1
0.4
0.2
0
-0.2
-0.4
0.6
b
WT
donor
WT enriched
KO
KO enriched
398
31
5
8
1
20
21
0
2
43
15
6
3
1
284
8
2
4
32
21
1
1
6
59
4
10
3
9
5
67
4
c
Class
Bacteroidia
Clostridia
Bacilli
Erysipelotrichi (FDR 0.0498, 0.2-fold)
Others(<0.5%)
Deferribacteres
Deltaproteobacteria
Betaproteobacteria (FDR 0.0869, 0.5-fold)
Unclassified
4C0d-2 (FDR <0.0869, 1.2-fold)
Verrucomicrobiae
Actinobacteria
Alphaproteobacteria
Gammaproteobacteria
Methanobacteria
Spirochaetes
Methanomicrobia
outlier
donor
WT
KO
standard microbiota
WT
KO
enriched microbiota
1
0
-1
-2
d
Order
Bacteroidales
Clostridiales
Lactobacillales
Erysipelotrichales (FDR 0.0546, 0.2-fold)
Others(<0.5%)
Deferribacterales
Burkholderiales
Desulfovibrionales
Unclassified
YS2
Verrucomicrobiales
Bifidobacteriales
RF32
Enterobacteriales
Turicibacterales
Methanobacteriales
Spirochaetales
Methanomicrobiales
SHA-98
Thermoanaerobacterales
Methanosarcinales
outlier
donor
WT
KO
standard microbiota
WT
KO
enriched microbiota
1
0
-1
-2

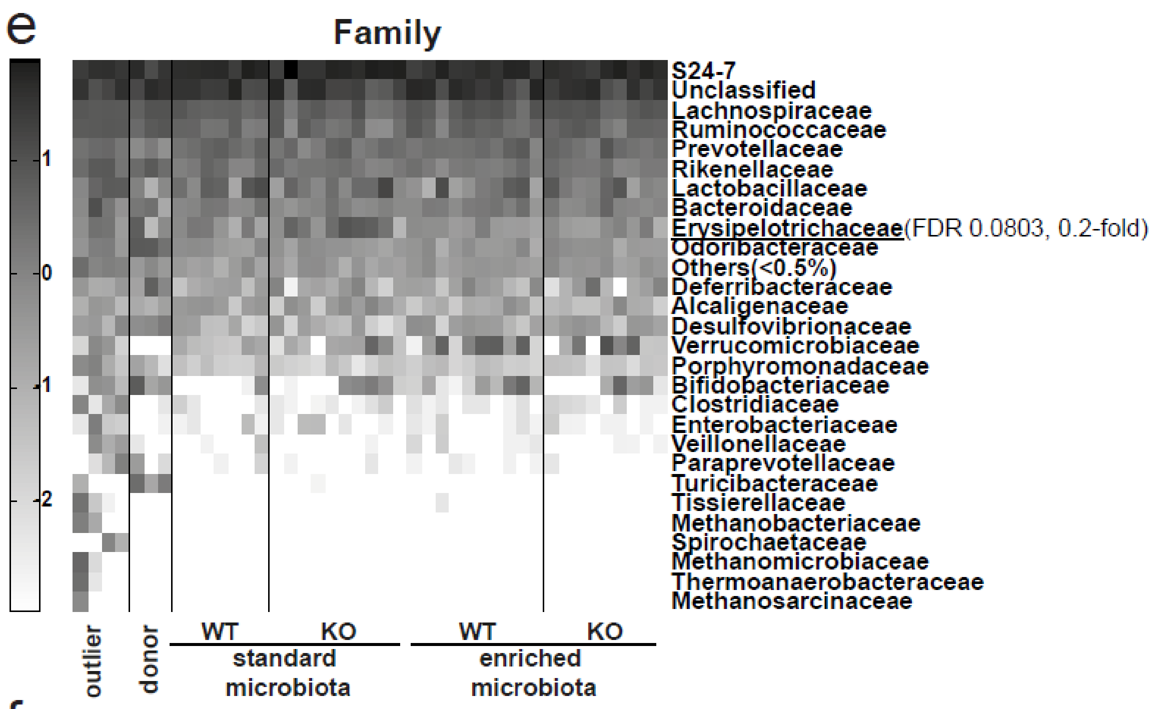
e
Family
1
0
-1
-2
S24-7
Unclassified
Lachnospiraceae
Ruminococcaceae
Prevotellaceae
Rikenellaceae
Lactobacillaceae
Bacteroidaceae
Erysipelotrichaceae(FDR 0.0803, 0.2-fold)
Odoribacteraceae
Others(<0.5%)
Deferribacteraceae
Alcaligenaceae
Desulfovibrionaceae
Verrucomicrobiaceae
Porphyromonadaceae
Bifidobacteriaceae
Clostridiaceae
Enterobacteriaceae
Veillonellaceae
Paraprevotellaceae
Turicibacteraceae
Tissierellaceae
Methanobacteriaceae
Spirochaetaceae
Methanomicrobiaceae
Thermoanaerobacteraceae
Methanosarcinaceae
outlier
donor
WT KO
standard microbiota
WT KO
enriched microbiota

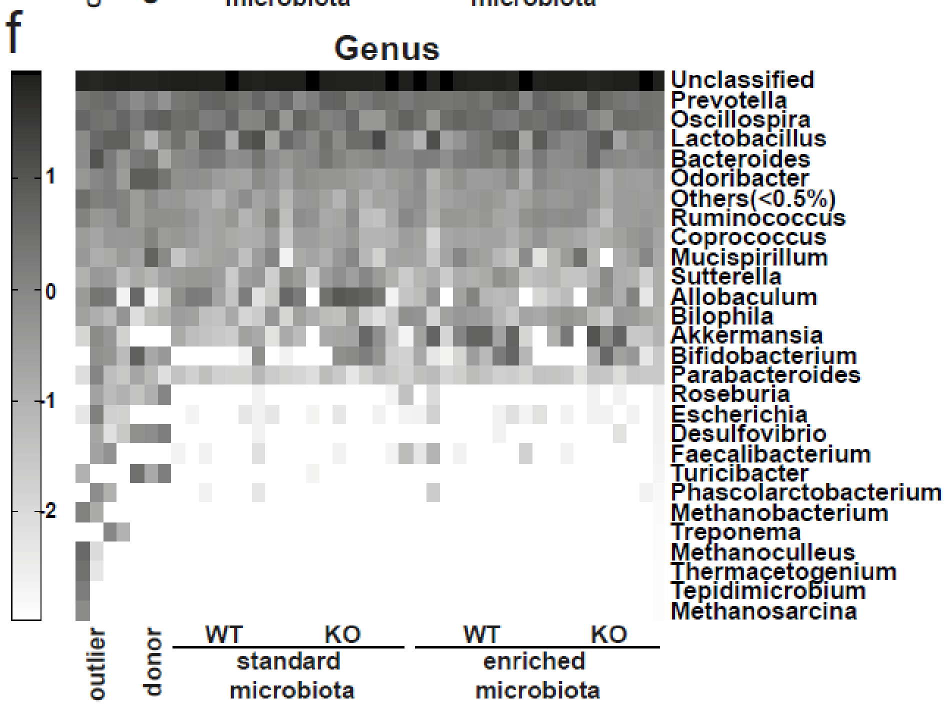
f
Genus
1
0
-1
-2
Unclassified
Prevotella
Oscillospira
Lactobacillus
Bacteroides
Odoribacter
Others(<0.5%)
Ruminococcus
Coprococcus
Mucispirillum
Sutterella
Allobaculum
Bilophila
Akkermansia
Bifidobacterium
Parabacteroides
Roseburia
Escherichia
Desulfovibrio
Faecalibacterium
Turicibacter
Phascolarctobacterium
Methanobacterium
Treponema
Methanoculleus
Thermacetogenium
Tepidimicrobium
Methanosarcina
outlier
donor
WT KO
standard microbiota
WT KO
enriched microbiota

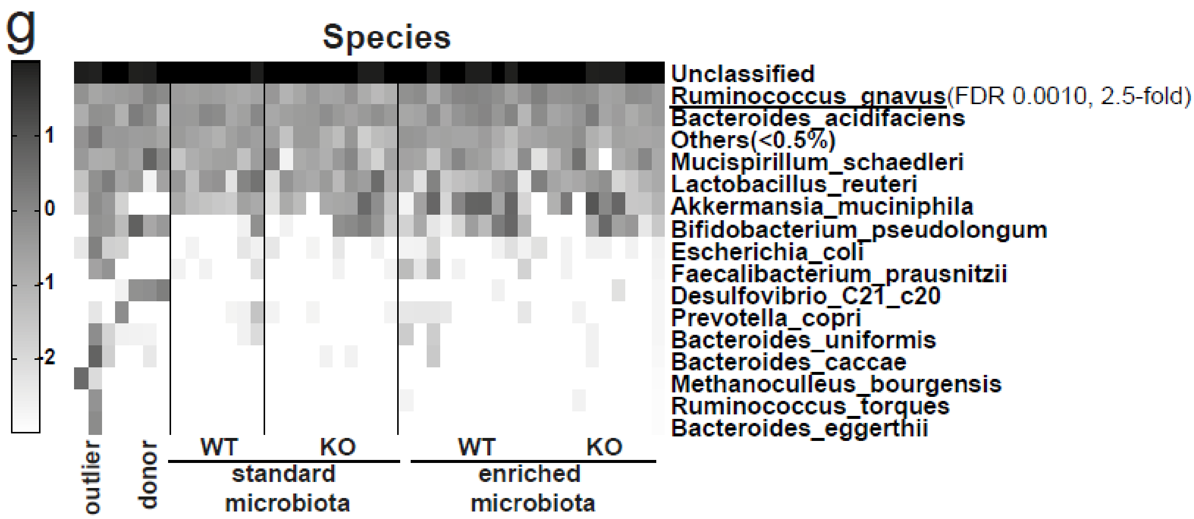
g
Species
1
0
-1
-2
Unclassified
Ruminococcus_gnavus(FDR 0.0010, 2.5-fold)
Bacteroides_acidifaciens
Others(<0.5%)
Mucispirillum_schaedleri
Lactobacillus_reuteri
Akkermansia_muciniphila
Bifidobacterium_pseudolongum
Escherichia_coli
Faecalibacterium_prausnitzii
Desulfovibrio_C21_c20
Prevotella_copri
Bacteroides_uniformis
Bacteroides_caccae
Methanoculleus_bourgensis
Ruminococcus_torques
Bacteroides_eggerthii
outlier
donor
WT KO
standard microbiota
WT KO
enriched microbiota

Figure S8: Analysis of microbiome transfer. a Principle Component Analysis based on OTU abundance in stool samples. Replicate groups are color-coded. Alpha-diversity outliers are *-indicated. Full list of 42 principal component values by sample is attached in Additional file 20: Table S19. **b** Venn diagram of detected OTUs per replicate group. OTUs not transferred from donor to recipients are marked in red. Candidate OTUs for enhanced colitis response and *Smarcad1*-mediated susceptibility are marked green. Additional file 23: Table S22 lists all OTUs, green and red sublists indicated accordingly. **c-g** Heat maps of log10 transformed OTU abundance per phylogenetic level: class (**c**), order (**d**), family (**e**), genus (**f**) and species (**g**). Rows sorted by average abundance. Phylogenetic terms significantly different between initial and enriched microbiota (FDR<0.1, Wilcox test, n=20, outliers not excluded) are indicated with FDR and fold changes (enriched/initial). Terms shown in Fig. 7d are underlined.

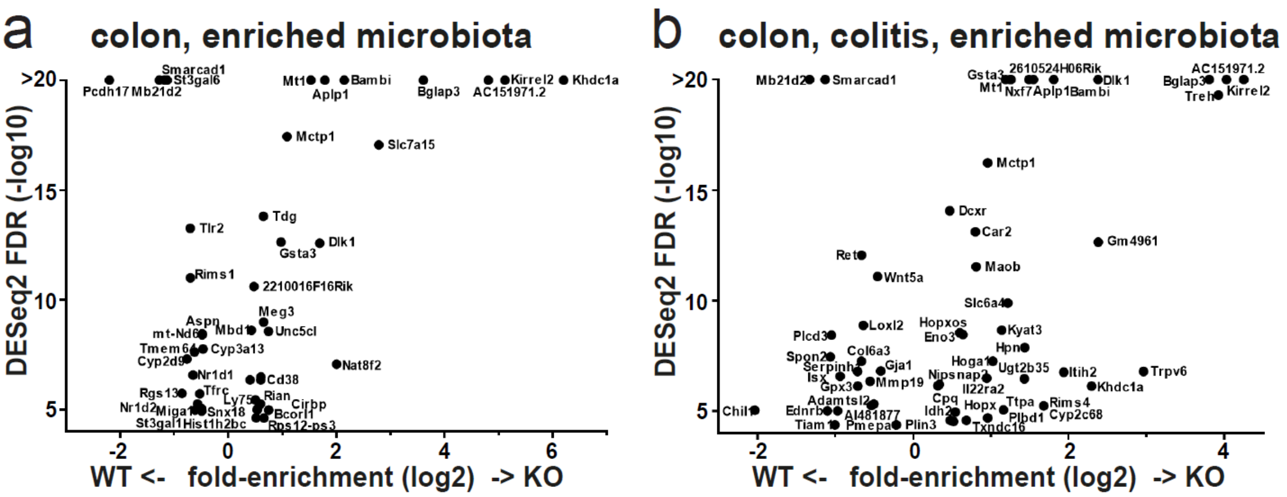

Figure S9: Differentially expressed genes in WT/ *Smarcad1*-KO in enriched microbiota samples before and during colitis. Significantly up/down-regulated genes (DESeq2 test with cutoff FDR<0.05) in whole colon tissue RNA-seq experiments. X-axis: *Smarcad1*-KO/WT log(2) fold-enrichment of reads per transcript. Y-axis: -log(10) DESeq2 FDR. High FDR values capped at indicated maximal Y-values for visualization. For additional data and annotations see Additional file 11: Table S10, and Additional file 12: Table S11.

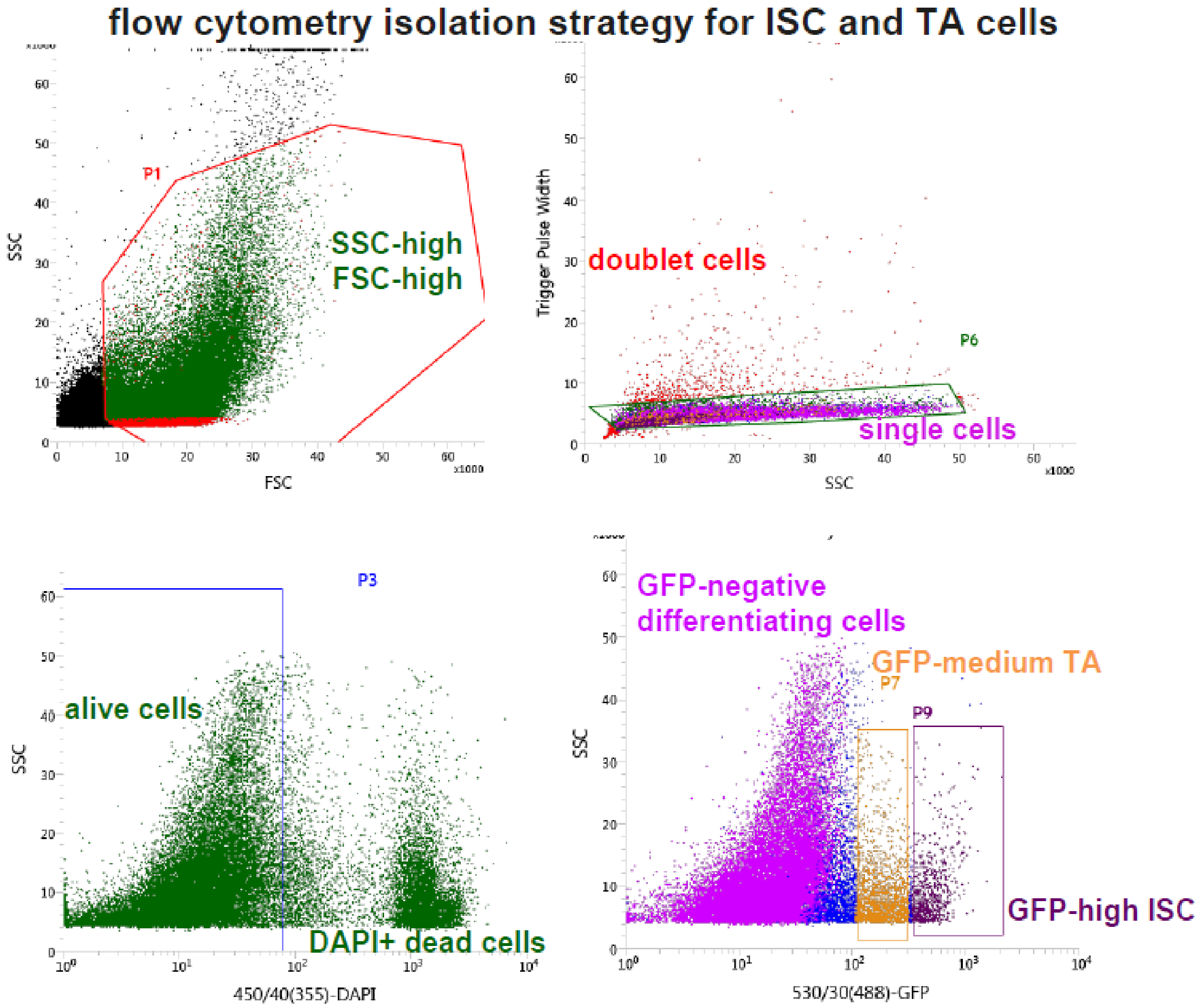

Figure S10: Flow sorting of ISC and TA for RNA-isolation. Crypt cell suspension is gated for cell shape and size via side scatter (SSC) and forward scatter (FSC) signals to discard cell aggregates and debris. Alive cells are gated as DAPI-low signal. *Lgr5*-GFP-high ISC and *Lgr5*-GFP-medium TA are sorted into lysis buffer simultaneously based on GFP-signal intensity.

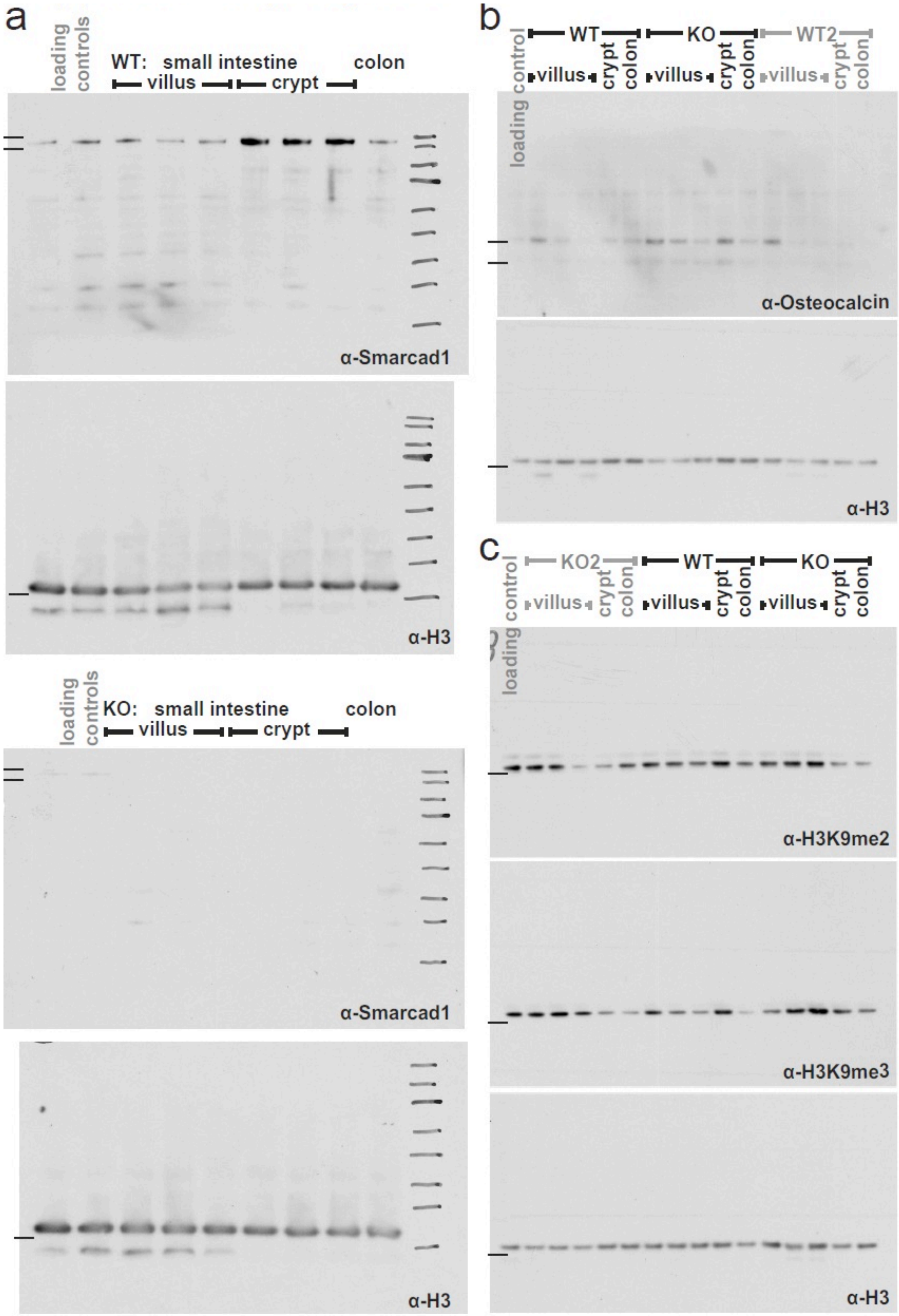
a
loading controls
WT: small intestine
villus
crypt
colon
α-Smarcad1
α-H3
loading controls
KO: small intestine
villus
crypt
colon
α-Smarcad1
α-H3
b
loading control
WT
KO
WT2
villus
crypt
colon
α-Osteocalcin
α-H3
c
loading control
KO2
WT
KO
villus
crypt
colon
α-H3K9me2
α-H3K9me3
α-H3

Figure S11: Uncropped Western blots. Full size scans of Western blot films. Blots shown in Fig. 1f, g (**a**), Additional file 1: Fig. S3c (**b**) and Additional file 1: Fig. S4a (**c**). Samples and inter-gel loading controls not shown in main text figures are greyed out. Relative molecular weights are indicated on the left according to Protein ladder. Smarcad1 blot: 140 kDa, 115 kDa. Osteocalcin blot: 25 kDa, 20 kDa and 15 kDa. H3 and H3k9me2/3 blots: 15 kDa.
