# Supplementary material for: Smarcad1 mediates microbiota-induced inflammation in mouse and coordinates gene expression in the intestinal epithelium
Source: Genome Biol. 2020 Mar 11;21:64. doi: 10.1186/s13059-020-01976-7 (PMC7065452; doi:10.1186/s13059-020-01976-7)
Supplement: Supplementary file 25 — Additional file 25: Protocol S1. ImageJ script and Excel annotation for the distance quantification of EdU signals on fluorescent images of the intestine as shown in Additional file 1: Fig. S1e-f. Related example files and Excel script in Additional file 24 Table S23. [file 13059_2020_1976_MOESM25_ESM.pdf]

Protocol S1

Calculations of EdU signal and distance to crypt bottom

1. ImageJ Macro

This macro allows to calculate the distance of Edu signal from the bottom of intestinal crypts. First, it will ask you to manually select the bottom of the crypts. This position array will be saved in “source picture name”.czi_crypts. Then, it will measure the Edu signal and DAPI signal yielding two files with positions of nuclei and corresponding EdU-signal intensities, named “source picture name”.czi_DAPI and “source picture name”.czi_Edu.

If the output of the macro is insufficient (low number of nuclei identified), “Find Maxima” in line 79 of the macro might need adjustment depending on noise levels.

After running the macro, in the original folder 2 new folders are created:

- ROI
- Results

The “Results” folder contains all files necessary for the follow-up distance analysis in excel.

Macro: Edu_in_Crypts.ijm

```
// by Hanneke Okkenhaug 21Jul2016
//All images have labelling of nuclei with DAPI and proliferation is marked with EdU.
// to analyse EdU intensity in the crypts and as a function of distance to the bottom of the crypt.

macro Measuring_EdU_in_Crypts{
dir = getDirectory("Select directory with files");
list = getFileList(dir);
setBatchMode(false);
File.makeDirectory(dir+"Results");
dir2 = dir+File.separator+"Results";
File.makeDirectory(dir+"ROIs");
dir3 = dir+File.separator+"ROIs";

for (m=0; m<list.length; m++){
{
            showProgress(m/list.length);
                        open(dir+list[m]);

title = getTitle();

//Opening your images and splitting the channels

myImageID = getImageID();
                                                    //remember the image
title = getTitle();
```

```
name=File.nameWithoutExtension();
run("Set Measurements...", "mean centroid redirect=None decimal=2");

run("Split Channels");
selectWindow("C1-"+title);
//run("Brightness/Contrast...");
run("Enhance Contrast", "saturated=0.35");

//Finding coordinates for the bottom of the crypts
//and saving the ROIs as a point selection

setTool("point");
                                                    //Point tool
run("Point Tool...", "type=Hybrid color=White size=[Extra Large] label");

waitForUser("Use Shift Click to select the bottom of the crypts you want to analyse");
                                          //wait for user action
selectWindow("C1-"+title);
                                                                        //make sure we have the same foreground
image again
titleD = getTitle();

s = selectionType();
if( s == -1 ) {
   exit("There was no selection.");
} else if( s != 10 ) {
   exit("The selection wasn't a point selection.");
} else {
   roiManager("Add");

Overlay.addSelection;

          //This bit creates the lines between the points
run("Measure");
 }

selectWindow("Results");
saveAs("txt", dir2+File.separator+title+"_crypts.xls");
run("Close");

selectWindow("ROI Manager");
roiManager("Select", 0);
selectWindow("C1-"+title);
saveAs("Selection", dir3+File.separator+title+"_roiCRYPTS.roi");
selectWindow("ROI Manager");
run("Close");              }
```

```
//Finding cells

selectWindow("C1-"+title);
run("Duplicate...", " ");


run("Find Maxima...", "noise=15 output=[Single Points]");
run("Options...", "iterations=1 count=1 black pad do=Dilate");

run("Set Measurements...", "mean min centroid integrated decimal=2");
run("Analyze Particles...", "size=0-infinite pixel include add");


//Measuring the EdU signal
selectWindow("C2-"+title);
titleE = getTitle();
roiManager("Measure");

selectWindow("Results");
saveAs("txt", dir2+File.separator+title+"_EdU.xls");
run("Close");

//Measuring the DAPI signal
selectWindow("C1-"+title);
titleD = getTitle();
roiManager("Measure");

selectWindow("Results");
saveAs("txt", dir2+File.separator+title+"_DAPI.xls");
run("Close");


//Saving the ROIs for the nuclei analysed
                    roiManager("Deselect");
                              roiManager("Combine");
                              roiManager("Delete");
                              roiManager("Add");
                              roiManager("Save", dir3+File.separator+title+"_roiEDU.zip");
                              roiManager("Deselect");
                              roiManager("Delete");
                              selectWindow("ROI Manager");
                              run("Close");


          run("Close All");
          }
showMessage("The macro is finished.");
}
```

2. Excel analysis

ImageJ analysis yields the following information for each dot: X/Y position, mean signal intensity, and number of dots. Next, in Excel the distance from the bottom of the crypts can be calculated for each Edu dot.

The Supplementary table 16 contains “example file” data as retrieved from ImageJ analysis.
To implement it into distance analysis, copy paste the data points as follows:

Copy paste the file .czi_crypts into the “crypts” tab columns A-D.
Copy paste the files .czi_Edu and .czi_DAPI into the “nuclei” tab columns A-I (DAPI) and J-R (EdU).

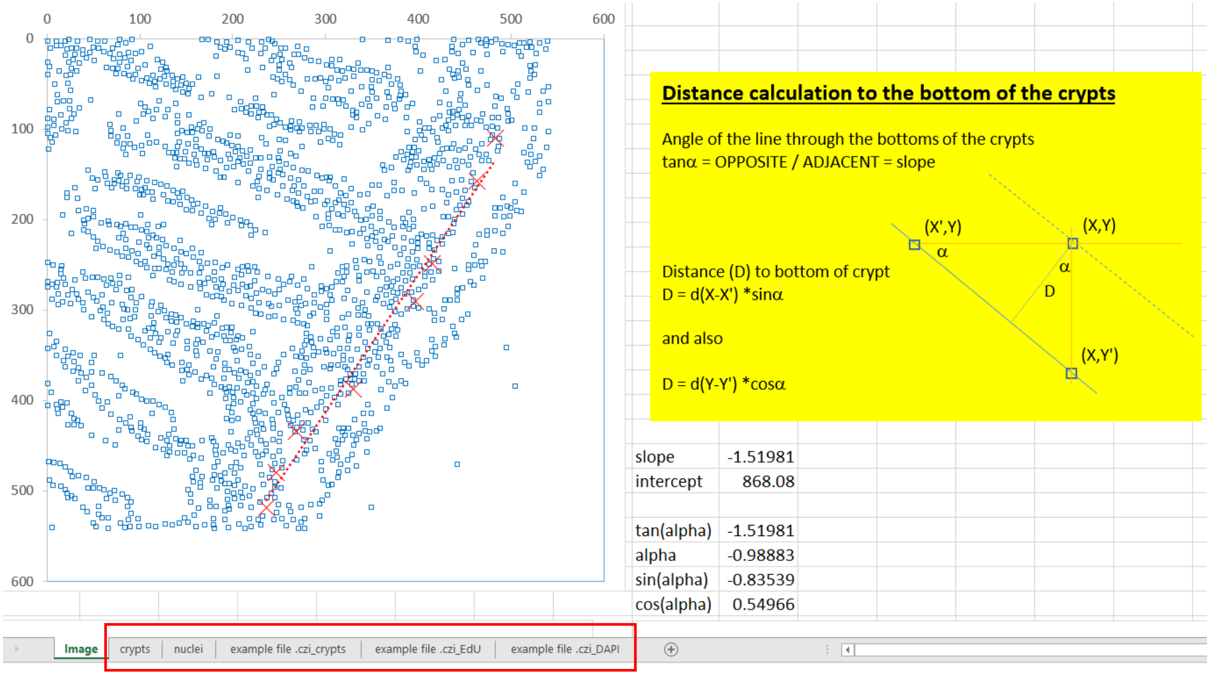

Screenshot of **Table S23**, tab “Image”. The red markers and trend line indicate the crypt bottomused for distance calculation. Blue markers indicate nuclei identified by the macro above. An explanation for the used distance calculation is highlited yellow. At the bottom the referred above data tabs and example files are indicated.

In the nuclei tab, the column “Distance” (yellow) will give you the distance from the bottom of the crypts.

This analysis is to be performed separately for each sample prior to statistical comparison of cohorts.
