# Supplementary material for: Smarcad1 mediates microbiota-induced inflammation in mouse and coordinates gene expression in the intestinal epithelium
Source: Genome Biol. 2020 Mar 11;21:64. doi: 10.1186/s13059-020-01976-7 (PMC7065452; doi:10.1186/s13059-020-01976-7)
Supplement: Supplementary file 26 — Additional file 26. Review history. [file 13059_2020_1976_MOESM26_ESM.docx]

Review History

**First round of review**

**Reviewer 1**

**Are you able to assess all statistics in the manuscript, including the appropriateness of statistical tests used?** No, I do not feel adequately qualified to assess the statistics.

**Comments to author:**

In the paper entitled "Smarcad1 mediates microbiota-induced inflammation by coordinating gene expression in the intestinal epithelium", Kazakevych et al. described a mouse model of Smarcard1 deletion in the intestinal epithelium. The authors found that the absence of Smarcad1 led to changes in gene expression, chromatin accessibility, and levels of H3K9me3. The authors also concluded that candidate members of the gut microbiome elicit a Smarcad1-dependent colitis response. Overall, the proposed model lacks mechanistic insights and their conclusion is not strongly supported by the results, since the intestinal phenotypes are not convincingly described and most data presented are correlative.

Specific comments:

The Fig. 1 describes Smarcad1 expression in the intestine without providing data how the KO is validated and characterized. The Suppl. Fig. 1C shows RNA-seq quantification of floxed exons 12-14 between WT and KO, but there are substantial variations within the KO samples and high levels of expression are still seen in the KO ISCs. Therefore, unfortunately, without validation and clear explanation, it is difficult to convince the readers.

The Fig. 2-5 describe changes in gene expression, chromatin accessibility, and H3K9 methylation. The use of organoids is not well explained. The analysis of hallmark genes linked to innate immunity and inflammation is superficial. This includes data describing H3K9 deposition only on several genes which is located either >10kb away from transcription start site or within the gene body. It is also unclear whether or not these changes link to chromatin accessibility.

The Fig. 6-7 describe the link to microbiome. The data regarding colitis susceptibility are not convincing in several ways. The authors should provide a histological characterization of the inflammation. The analysis is highly descriptive and the results are inconclusive.

**Reviewer 2**

**Are you able to assess all statistics in the manuscript, including the appropriateness of statistical tests used?** No, I do not feel adequately qualified to assess the statistics.

**Comments to author:**

This manuscript performs global genome analyses on intestinal epithelial cells lacking the Smarcad1. Not surprisingly, mice that do not express this chromatin remodeling gene exhibit alterations in gene expression and chromatin accessibility/histone methylation. The authors then attempt to link Smarcad1 to microbiota during DSS colitis.  However the data and analyses included in the current form of this manuscript do not provide convincing support for a causative regulatory link with microbiota.

1. Authors propose this work demonstrates "causative" links between microbiota. This is an overstatement. Protection against DSS-induced colitis when distinct flora is added is not sufficient to conclude this. Authors would need to demonstrate that gene expression and chromatin changes observed with Smarcad1 deletion in epithelial cells are dependent on the microbiota (ie- no longer occur when flora depleted/absent; or when distinct microbiota is reconstituted)

2. Despite performing extensive global analyses, the data presentation is very limited and lacks depth. The authors should include figures showing how the ChIP-seq and ATAC-seq data correlates with RNA-seq. Only selected targets are shown without functional rational supporting why these targets should be highlighted. Non-biased analyses are needed to assess how chromatin alterations in Smarcad1 knockout cells globally correlate with differential expression.

3. Authors highlight that several genes that exhibit increased expression in Smarcad1 knockouts are characterized by decreased H3K9Me3 levels in the promoter region. This mark typically characterizes active transcription. The authors need to address this discrepancy.

4. It is not clear whether upregulated genes are direct targets of Smarcad1. The authors acknowledge a technical limitation of performing ChIP for Smarcad1, however if these are direct targets, then is the ATAC-seq prediction would be increased accessibility in the knockouts. The data does not suggest many DE targets exhibit this pattern.

5. Given that there is no knowledge of functional significance of Bglap3 in intestinal epithelium, DSS colitis, or in relation to microbiota, it does not make sense to single this gene out in the analyses (Figure 2, Supplemental Fig. S2). Adding global comparisons (ie. expression heatmap and pathway analyses with stats in Fig 2, correlation of all DE upregulated with ChIP-seq/ATAC-seq data in Fig 4 and Fig 5) would provide more useful data for demonstrating the scope of Smarcad1 regulation in the epithelium.

6. Unclear why authors needed to Venn analyses in Fig. 3 to overlap between sorted cells and crypts; or different SI regions and SI organoids. Its hard to believe that the critical shared genes impacted by loss of Smarcad1 in these different cell preps are limited to those highlighted in the Venn comparisons.

7. Impossible to draw conclusions on DSS data (Fig. 7C) without accounting for differential expression of targets in the naïve state. Should perform expression analyses in DSS in relation to naïve expression of the same genotype to determine if DSS responsive genes in WT mice are still induced/repressed with DSS in KOs,  and how this differs with altered microbiota. Analyses similar to Fig 6C and 6D should be included for naïve colon IECs in parallel with DSS colon IECs.

8. Its very confusing why Smarcad1 knockouts would not be protected with 2.5% DSS given that this colitis is dependent on the microbiota. If authors propose it is due to a few specific microbes in the 'enriched micorbiota' along with 1% DSS, then this should be experimentally proven.  What is the effect of 1% DSS without an "enriched microbiota"? This important control expt is lacking.

9. Isolation of homogenous or similar epithelial populations from the WT and KOs would be difficult during DSS given the difference shown in phenotype. How were these cells purified from the DSS model? Were mice being compared to littermate controls (ie- cre - and cre + floxed mice from same breeders) for microbiota analyses and DSS. This is essential for drawing any microbiota-related conclusions given that litters can have marked variability in composition and DSS response.

10. The interpretation of the enriched microbiota studies is not substantiated. Loss of Smarcad1 could lead to more protective epithelial barrier so protection observed with 1% DSS colitis could still be independent of microbiota.

11. Fig. Supl 1C: Why is the background signal for Smarcad1 so high in KOs?

**Authors Response**

**Point-by-point responses to the reviewers’ comments:**

**Reviewer #1:
Specific comment 1:**

“The Fig. 1 describes Smarcad1 expression in the intestine without providing data how the KO is validated and characterized. The Suppl. Fig. 1C shows RNA-seq quantification of floxed exons 12-14 between WT and KO, but there are substantial variations within the KO samples and high levels of expression are still seen in the KO ISCs. Therefore, unfortunately, without validation and clear explanation, it is difficult to convince the readers.”

**Our response:**

“We have now performed the quantification more carefully, only selecting deleted exons. The new Figure S1c shows now convincingly that Smarcad1 is deleted in the Vil-cre mice. The western blots shown in Figures 1f, g and quantified in Fig. S1b also clearly show loss of Smarcad1 protein expression upon excision by Vil-cre.

**Specific comment 2**
The Fig. 2-5 describe changes in gene expression, chromatin accessibility, and H3K9 methylation. The use of organoids is not well explained.

**Our response:**

We clarified the use of organoids, writing:

“We also performed mRNA-seq on small intestinal organoids isolated from mice where *Smarcad1* had been deleted and controls, to assess microbiota- and immune system independent gene expression (**Fig. 2f**).” and

“Interestingly, *Mt1* is not significantly upregulated in the small intestinal organoid culture upon *Smarcad1*-KO, indicating that this upregulation may depend on some external cue, such as niche, microbiota or immune cells (**Fig. 2**, **Fig. S2**.”

**Specific comment 3:**

“The analysis of hallmark genes linked to innate immunity and inflammation is superficial. This includes data describing H3K9 deposition only on several genes which is located either >10kb away from transcription start site or within the gene body. It is also unclear whether or not these changes link to chromatin accessibility.”

**Our response:**

We have now performed better bioinformatic analysis, including testing the link between changes in H3K9me3, chromatin accessibility and gene expression. Please, see our response to reviewer 2 (comment 2).

**Specific comment 4:**

“The Fig. 6-7 describe the link to microbiome. The data regarding colitis susceptibility are not convincing in several ways. The authors should provide a histological characterization of the inflammation. The analysis is highly descriptive and the results are inconclusive.”

**Our response:**

We now provide a histological characterization of the inflammation (new Fig. 6c, d and Fig. S7), visualisation the invasion of the epithelium by neutrophils/ monocytes during the DSS colitis. Our new gene ontology analysis of DSS upregulated genes (Fig. S6g)

also confirms an inflammatory reaction.

**Reviewer #2:**

**Comment 1**
“Authors propose this work demonstrates "causative" links between microbiota. This is an overstatement. Protection against DSS-induced colitis when distinct flora is added is not sufficient to conclude this. Authors would need to demonstrate that gene expression and chromatin changes observed with Smarcad1 deletion in epithelial cells are dependent on the microbiota (ie- no longer occur when flora depleted/absent; or when distinct microbiota is reconstituted).”

**Our response:**

We performed the DSS experiment with mice without enriched microbiota and show that these mice do not show the Smarcad1 dependent colitis response seen after microbiota enrichment, new Fig. 6a. This strengthens the link between Smarcad1 function and a specific microbiome.

This reviewer may have misunderstood our take home messages. We do not wish to imply that Smarcad1 is part of a direct response cascade to the microbiota, our data do not show this. Instead Smarcad1 is regulating a number of genes involved in anti-microbial defence. Indeed, we show that gene expression changes linked to anti-microbial defence, such as upregulation of lysozyme, are observed in gut organoid culture, where no microbiota is present. We clarified our take home messages in the abstract, discussion and conclusion, writing:

abstract:

“Our study highlights the role of the chromatin remodeling machinery in intestinal epithelial cells in the colitis response and shows how a highly conserved chromatin remodeling factor has a distinct role in anti-microbial defense**.** Our work flags up the potential of microbial species as pharmacological and probiotic targets in the context of inflammatory diseases.”

Discussion:

“We did not find evidence that deletion of *Smarcad1* affects intestinal cell turnover or epithelial barrier function, but rather we found that it leads to changes in gene expression that are consistent with a protective effect. In the steady state condition, we found that *Smarcad1*-KO promoted expression of several genes linked to innate immunity, such as Toll-like receptor *Tlr4*, anti-bacterial peptides and other factors controlling inflammatory responses such as *Lyz1*. One gene that is highly overexpressed upon *Smarcad*1-KO in steady-state and colitis is *Mt1*, coding for metallothionein. Metallothionein has been reported to protect against colitis in mouse models, but its role requires further investigation [52–54,27,55,56]. This suggests that Smarcad1 is normally involved in a pathway that dampens an innate immunity response and upon its deletion the intestinal epithelium may already be primed to deal with a microbial challenge during the DSS treatment. On DSS treatment, the deletion of *Smarcad1* leads to specific changes in gene expression, consistent with the reduced colitis response. Together, our data suggest that this chromatin remodelling factor orchestrates the expression of genes involved in an inflammatory response in the gut. As we made these observations with an intestinal epithelium specific deletion of *Smarcad1*, our observations underscore the importance of the intestinal epithelial tissue and innate immunity-linked processes in mediating a colitis response.”

Conclusion:

“We identify candidate bacterial species crucial for the phenotype severity of colitis, highlighting their potential as targets for pharmacological and probiotic treatment of intestinal inflammatory diseases.”

**Comment 2**
Despite performing extensive global analyses, the data presentation is very limited and lacks depth. The authors should include figures showing how the ChIP-seq and ATAC-seq data correlates with RNA-seq. Only selected targets are shown without functional rational supporting why these targets should be highlighted. Non-biased analyses are needed to assess how chromatin alterations in Smarcad1 knockout cells globally correlate with differential expression.
**Our response:**

We have now performed the requested analysis and show the results of such analyses in new figures. We also introduced additional, new H3K9me3 ChiP-seq data and analysis of colon epithelium (previously we only showed small intestine, new Fig. 4).

Overall, the result show that there is a significant link between alterations in H3K9me3 and changes in gene expression (new Fig. 3 d, e; new Fig. 4). There is also a significant link between changes in chromatin accessibility and changes in H3K9me3 (new Fig. 5b).

**Comment 3**

“Authors highlight that several genes that exhibit increased expression in Smarcad1 knockouts are characterized by decreased H3K9me3 levels in the promoter region. This mark typically characterizes active transcription. The authors need to address this discrepancy.”

Our response:

H3K9me3 is a paradigm repressive mark (the reviewer probably thinks of H3K4me3, which is a paradigm activating mark). Thus, there is no discrepancy.

**Comment 4**

It is not clear whether upregulated genes are direct targets of Smarcad1. The authors acknowledge a technical limitation of performing ChIP for Smarcad1, however if these are direct targets, then is the ATAC-seq prediction would be increased accessibility in the knockouts. The data does not suggest many DE targets exhibit this pattern.
Our response:

We agree that we only observed few changes in gene accessibility by our ATAC-seq approach.

We now write in the section describing the ATAC-seq data:

We only observed few changes in gene accessibility by our ATAC-seq approach. While this may be due to technical limitations, it might reflect the biology of the intestinal epithelium. In this context, it is interesting that a previous study found that changes in gene expression during intestinal cell maturation do not involve dramatic changes in chromatin accessibility  [Broadly permissive intestinal chromatin underlies lateral inhibition and cell plasticity.](https://www.ncbi.nlm.nih.gov/pubmed/24413398) Kim TH, Li F, Ferreiro-Neira I, Ho LL, Luyten A, Nalapareddy K, Long H, Verzi M, Shivdasani RA. Nature. 2014 Feb 27;506(7489):511-5. doi: 10.1038/nature12903. Epub 2014 Jan 12.

**Comment 5**

Given that there is no knowledge of functional significance of Bglap3 in intestinal epithelium, DSS colitis, or in relation to microbiota, it does not make sense to single this gene out in the analyses (Figure 2, Supplemental Fig. S2). Adding global comparisons (ie. expression heatmap and pathway analyses with stats in Fig 2, correlation of all DE upregulated with ChIP-seq/ATAC-seq data in Fig 4 and Fig 5) would provide more useful data for demonstrating the scope of Smarcad1 regulation in the epithelium.

**Our response:**

Additional bioinformatic analysis of H3K9me3, chromatin accessibility and gene expression are now added in new Fig. 3-5. *Bglap3*, together with only few other genes, is consistently upregulated on *Smarcad1*-KO and therefore constitutes a potent candidate for the downstream function of Smarcad1. This justifies a closer look into *Bglap3* expression, localization and functional role (Fig. S3). We agree that *Bglap3* is not the key topic of this paper and adjusted the discussion section accordingly, removing the paragraph about it. Other genes, consistently up-/downregulated and known to be involved in the colitis response, such as *Lyz1* and *Mt1* on *Smarcad1*-KO are now discussed in more detail:

See also response to comment 1, reviewer 2.

**Comment 6**

Unclear why authors needed to Venn analyses in Fig. 3 to overlap between sorted cells and crypts; or different SI regions and SI organoids. Its hard to believe that the critical shared genes impacted by loss of Smarcad1 in these different cell preps are limited to those highlighted in the Venn comparisons.

Our response:

We placed this figure now as Fig. S2 in the supplementary data file, as it highlights the most robust gene expression changes on *Smarcad1*-KO. RNA-seq data and the bioinformatic analyses are generally subject to variation in sequencing quality, depth and quantification noise due to arbitrary statistical cut-offs. While each experiment has value in itself, the overlapping gene lists shown as Venn diagrams demonstrate the most robust DEG.

**Comment 7**

Impossible to draw conclusions on DSS data (Fig. 7C) without accounting for differential expression of targets in the naïve state. Should perform expression analyses in DSS in relation to naïve expression of the same genotype to determine if DSS responsive genes in WT mice are still induced/repressed with DSS in KOs, and how this differs with altered microbiota.  Analyses similar to Fig 6C and 6D should be included for naïve colon IECs in parallel with DSS colon IECs.

**Our response:**

Our work is not targeting microbiome-induced gene expression changes, but uses the microbiome enrichment to facilitate the DSS-induced colitis model. Without the enrichment there no clear colitis response (new Fig. 6a). Analysis shown in what is now Fig. 8 specifically targets gene clusters behaving differentially between the WT and the KO background in response to colitis. The same experimental setup without microbiota enrichment would be technically possible, but we do not think it would add value to the data analysis, as no full colitis response would be triggered (see Fig. 6a). Therefore, this would probably constitute a snapshot between the two shown states of healthy, microbiota enriched gene expression and the microbiota enriched and DSS-induced colitis.

**Comment 8**

“Its very confusing why Smarcad1 knockouts would not be protected with 2.5% DSS given that this colitis is dependent on the microbiota. If authors propose it is due to a few specific microbes in the 'enriched micorbiota' along with 1% DSS, then this should be experimentally proven.  What is the effect of 1% DSS without an "enriched microbiota"? This important control expt is lacking.”

Our response:

We thank the reviewer for the valuable suggestion to perform the experiment without enriched microbiota in 1% DSS. We agree with the reviewer that showing the 2.5% DSS experiment is confusing, and replaced it with a more straight-forward 1% DSS control experiment which showed no DSS mediated weight loss, unlike with the enriched microbiota (new Fig. 6a, Fig. S6a,c).

The next logical step in this project would be to test which of the candidate bacterial species are responsible for the colitis. We cannot inoculate mice specifically with our prime candidate colitogenic bacteria species, TM7_CW040_F16, as this has yet not been isolated.

**Comment 9**

Isolation of homogenous or similar epithelial populations from the WT and KOs would be difficult during DSS given the difference shown in phenotype. How were these cells purified from the DSS model?

Our response:

Separate cell types were not isolated from colitis experiments, here full tissue was processed for RNA-seq. We did this analysis primarily to monitor colitis progression using molecular tools and to identify *Smarcad1* specific response genes. A number of DEG was identified in this analysis (new Fig. 8). We analyse and discuss the bias generated by non-epithelial changes in the tissue as follows:

“Stainings for Adamts1, Adamts5 and Bmp1 showed similar distribution patterns as the MPO-staining, indicating that these proteases are contributed not by the colon epithelium, but by invading neutrophils/ monocytes on colitis-induction (**Fig. S7**).”

Were mice being compared to littermate controls (ie- cre - and cre + floxed mice from same breeders) for microbiota analyses and DSS. This is essential for drawing any microbiota-related conclusions given that litters can have marked variability in composition and DSS response.

**Our response:**

Yes, all experiments were performed on littermates. Microbiome enrichment was performed on littermates sharing cages to guarantee comparable microbiota enrichment. This was confirmed by our analysis shown in Fig. 7b, demonstrating no genotype-related clustering of the microbiomes in the initial or enriched states.

**Comment 10**

The interpretation of the enriched microbiota studies is not substantiated. Loss of Smarcad1 could lead to more protective epithelial barrier so protection observed with 1% DSS colitis could still be independent of microbiota.

**Our response:**

We tested the barrier function of the epithelium of the Smarcad1-KO mice (using a standard method with FITC-dextran, new Fig. S1 g). This showed no change in barrier function in the steady-state condition. We write in Results:

“We did not detect changes in the barrier function by the FITC-dextran assay (**Fig. S1g**).”

**Comment 11**

Fig. Supl 1C: Why is the background signal for Smarcad1 so high in KOs?

**Our response:**

We re-quantified the data in a better way, specifically selecting excised exons. This led to a more convincing picture, see our response to comment 1, reviewer1.

**Second round of review**

**Reviewer 1**

The manuscript titled "Smarcad1 mediates microbiota-induced inflammation by coordinating gene expression in the intestinal epithelium" is an assessment of intestinal specific deletion of Smarcad1 in a mouse model of colitis. The authors have characterized changes in chromatin accessibility and histone H3K9me3, and have also conducted an analysis of gut microbiome in response to Smarcad1-dependent colitis. This study provides new insights into this mouse model but has a number of issues that limits its significance and weaken the strength of the conclusions. These limitations are related to the experimental design and to the conclusions drawn by the author. In addition, the observation of specific bacterial species associated with colitis response is interesting, but the lack of functional validation and the lack of relevance to human IBDs limit the impact of this manuscript.

Specific comments
1.     Recently, Sachs et al. provided compelling evidence on how SMARCAD1 binding sites are enriched at heterochromatin regions in mouse ESCs( Nat. Communication 2019; 10: 1335). Authors should discuss how their ChIP antibodies and ChIP-seq protocols are related or differed.
2.     It appears that deletion of Smarcad1 led to opposing effects of H3K9me3 changes in small intestine vs. colon. As pointed out above, present study did not examine SMARCAD1 occupancy, thus it is not possible to determine whether Smarcad1 is directly responsible for the observed chromatin and gene expression changes, or mere by-products of other events.
3.     The mixing of decreased and increased H3K9me3 changes substantially confounds results. It is not appropriate to mix them as they have different function on gene expression.
4.     Unfortunately, most important functional studies are missing. Underlying mechanism how deletion of Smaracd1 affects anti-microbial defense is not clarified in the study.
5.     The analysis of colitis is also a substantial weakness as the only measurement was body weight loss. A much more thorough and objective analysis including detailed histological characterization should be conducted.  
6.     The characterization of bacterial species responsible for Smarcad1-depdent colitis is not stringent enough. Is there a common mechanism by which TM7 triggers colitis response? Have the authors tried colitis-prone microbiome from other mouse facilities besides the University of York? Whether and to what extent the findings from the present study are relevant for understanding the mechanistic basis of human inflammatory diseases?
7.     Overall, this is a very descriptive study and the statements related to Smarcad1 and microbiota-induced inflammation are overstated given the lack of convincing data to support the conclusions drawn by the authors.

**Reviewer 2**

The authors have adequately addressed most comments.

1. The title should be modified to reflect the more accurate interpretation that they now use in the abstract/text (Rev 2, Comment 1). The original title indicates causation, but a title similar to below would be in line with the data presented.

Smarcad1 mediates microbiota-induced inflammation AND coordinates gene expression in the intestinal epithelium

2. The authors author include colon analyses for H3K9me3, and gene expression (new Fig. 4). This seems necessary given that DSS is a model of colitis. However, they note very little correlation between H3K9me3 and DEG. How do the authors interpret this finding and how do these DEGs relate to those genes observed during microbiota-enriched colitis that they describe in Fig 8?

**Authors Response**

**Point-by-point responses to the reviewers’ comments:**

**Reviewer #1** writes that our study “provides new insights into this mouse model” and “the observation of specific bacterial species associated with colitis response is interesting”. However, this referee wishes to strengthen the manuscript by addressing several comments:

**Specific comments**

**Comment 1.**      Recently, Sachs et al. provided compelling evidence on how SMARCAD1 binding sites are enriched at heterochromatin regions in mouse ESCs (Nat. Communication 2019; 10: 1335). Authors should discuss how their ChIP antibodies and ChIP-seq protocols are related or differed.

**Our response:**

We tried the same antibody and protocol as published in the Sachs et al., study, but could not obtain successful ChIP-seq results. We believe that the Sachs et al. study provides a plausible answer why this is the case: They show that Smarcad1 is much more highly expressed in embryonic stem cells than somatic type cells (Fig. 1a of this study), and, thus, signal-to-noise ratio would not be favourable for ChIP-seq of Smarcad1 from tissue. We write now in Discussion:

“*Unlike the study from Sachs et al. that document Smarcad1 binding by ChIP-seq [49], we have not been able to produce a Smarcad1 ChIP-seq profile employing same antibodies and protocols. This may relate to the much lower expression of Smarcad1 in somatic type cells compared to embryonic stem cells, as shown by the Sachs et al. study. Furthermore, the predominant localization of Smarcad1 in the stem and proliferative compartment of the intestinal epithelium is consistent with a global rather than locus specific role, e.g. maintenance of heterochromatin through replication [10].”*

**Comment 2.**      It appears that deletion of Smarcad1 led to opposing effects of H3K9me3 changes in small intestine vs. colon. As pointed out above, present study did not examine SMARCAD1 occupancy, thus it is not possible to determine whether Smarcad1 is directly responsible for the observed chromatin and gene expression changes, or mere by-products of other events.
**Our response:**

We found that deletion of Smarcad1 affected H3K9me3 over many sites in both small intestine and colon. However, we identified many more sites (MACS peaks) where H3K9me3 decreased in small intestine compared to colon epithelium (compare new Fig. 3d and 4c).

We write now (end of page 7):

*“In contrast to the small intestine, we did not observe globally a decrease of H3K9me3 over differentially expressed genes and that this mark generally increased over down-regulated genes (Fig. 4d). The observed difference between H3K9me3 over up-regulated genes in small intestine versus colon epithelium may relate to the fact that proportionally we observe more sites where H3K9me3 decreases in the small intestine compared to colon epithelium (compare new Fig. 3d and 4c).”*

And in Discussion (end of page 11):
*“We observed a notable difference in the number of sites where H3K9me3 decreases between the small intestine and colon epithelia, possibly linked to the histological differences in the cells we isolated for the analysis. We isolated crypts of both small intestine and colon tissue for the ChIP-seq analysis. Colon crypts contains proportionally more differentiated cells compared to small intestine crypts. Future analysis should unravel the role of Smarcad1 in H3K9me3 establishment and maintenance during differentiation”.*

**Comment 3.**      The mixing of decreased and increased H3K9me3 changes substantially confounds results. It is not appropriate to mix them as they have different function on gene expression.

**Our response:**

We have now revised the analysis shown in new Figures 3d, 4c and 5b, analysing overlaps between gene expression and H3K9me3 changes for decreased and increased H3K9me3 (Figures 3d, 4c) and overlaps between changes in accessibility (ATAC-seq) and H3K9me3 changes for decreased and increased H3K9me3 (Fig. 5b). We find there is a significant overlap between genes showing decrease in H3K9me3 and increase in expression in the small intestine epithelium (Fig. 3d). There is also a large overlap between genes that show a decrease in H3K9me3 in or nearby the gene and those that show an increase in accessibility (Fig. 5b). For the latter analysis, we changed the parameters for the overlap, exploring all genes that show changes in H3K9me3 AND accessibility within 5Kbp of the gene.  This revised and more meaningful approach consolidated our findings and improved overlap-analysis compared with our initial analysis. In the colon epithelium, there is a statistically significant overlap between genes that show increased H3K9me3 and decrease in expression (Fig. 4c, one needs to compare the number of genes where this overlap occurs with the total number of expressed genes).

**Comment 4.**      Unfortunately, most important functional studies are missing. Underlying mechanism how deletion of Smarcad1 affects anti-microbial defense is not clarified in the study.

Our response:

We found that deletion of *Smarcad1* causes upregulation of several genes that are known to protect against colitis or inflammation such as *Mt1* or *Bambi* and downregulation of genes that normally promote inflammation, such as *Tiam1*, even in the steady state condition. It is beyond of the scope of this manuscript to unravel which of these candidate factors or all of them in combination are involved in the protection against colitis, when we delete Smarcad1.

We are writing (Discussion, end of page 12 onwards):

*“We did not find evidence that deletion of Smarcad1 affects intestinal cell turnover or epithelial barrier function, but rather we found that it leads to changes in gene expression that are consistent with a protective effect. In the steady state condition, we found that Smarcad1-KO promoted expression of several genes linked to innate immunity, such as Toll-like receptor Tlr4, anti-bacterial peptides and other factors controlling inflammatory responses such as Lyz1. One gene that is highly overexpressed upon Smarcad1-KO in steady-state and colitis is Mt1, coding for metallothionein. Metallothionein has been reported to protect against colitis in mouse models, but its role in this process requires further investigation [53–55,27,56,57]. Another gene that is upregulated upon Smarcad1 deletion codes for Bambi. Bambi is a TGF-beta decoy receptor that dampens or blocks the activity of this cytokine, and thus controls inflammatory responses [30,58]. TGF-beta is involved in inflammatory responses, including in colitis [59]. This suggests that Smarcad1 is normally involved in a pathway that controls an innate immunity response and upon its deletion the intestinal epithelium may already be primed to deal with a microbial challenge during the DSS treatment. On DSS treatment, the deletion of Smarcad1 leads to specific changes in gene expression consistent with the reduced colitis response.”*

We have started to explore changes in protein levels of some of these candidate genes. However, we found that the lack of high quality antibodies, e.g. against Bambi, necessitates a dedicated effort that is beyond the scope of this manuscript. For example, an antibody against Bambi (~ 30 kDa) appears to detect increased expression of this protein in colon epithelium of Smarcad1 KO mice, but also detects a ~ 80 kDa protein that appears also over-expressed (see Figure below, shown only for the reviewers). It is not clear if this is a yet uncharacterised splice variant, a post-translationally modified Bambi or just cross-reactivity of the antibody with another protein.

**Figure: Bambi expression in colon epithelium extracts of Smarcad1 KO and control mice.** Shown are western blot analysis of extracts from 4 Smarcad1 KO mice (lanes 1-4) and two control mice (lanes 5,6). Molecular size in kDa is indicate at the left. Bambi normally migrates as a ~ 30 kDa protein (lower arrow). A larger protein is also detected (upper arrow).

**Comment 5.**      The analysis of colitis is also a substantial weakness as the only measurement was body weight loss. A much more thorough and objective analysis including detailed histological characterization should be conducted.

Our response:

We provide histological characterization, Fig. 6c and d, and Fig. S7, which shows distribution of MPO-positive cells (staining neutrophils and monocytes) in the intestinal epithelium before and during the DSS-treatment in KO and WT animals. This shows an increase in these cells during the DSS colitis and that there is a trend of less MPO^+^ cells invasion in the Smarcad1-KO epithelium. We also followed the response to the DSS treatment by monitoring stool consistence, blood in stool and appearance of animals (disease activity index, DAI), which also shows differences between KO and WT animals (Figures S6d and S6e, table S9, exp. 2).

Most importantly, our transcriptomic analysis (Fig. 8) provides an objective and powerful molecular characterization of the DSS-mediated colitis and the effect of Smarcad1 deletion in this process.

**We write:**

*“To explore this on a molecular level, we extracted mRNA from colon tissue of untreated mice and from mice after DSS treatment and performed transcriptome analysis by RNA-seq (****Fig. S9****,* ***Tables S10****,* ***S11****). We identified 3261 DEG on colitis induction in WT (wild type) mice (DESeq2 test, cut-off FDR<0.05,* ***Table S12****) and gene ontology analysis confirms a strong link of these genes to inflammatory responses (****Fig. S6g****). Most of these genes respond in the similar way to colitis on Smarcad1-KO (****Fig. 8a****). However, a subset of genes shows incomplete upregulation on colitis induction in Smarcad1-KO mice…” (End pf page 9)*

and

*“The transcriptome based analysis of the colitis response illustrated the up-regulation or shutdown of expression of many genes upon DSS treatment and shows that this transcriptional response was subdued for many genes in the intestine specific Smarcad1-KO mice” (End of page 10).*

**Comment 6.**      The characterization of bacterial species responsible for Smarcad1-depdent colitis is not stringent enough. Is there a common mechanism by which TM7 triggers colitis response? Have the authors tried colitis-prone microbiome from other mouse facilities besides the University of York? Whether and to what extent the findings from the present study are relevant for understanding the mechanistic basis of human inflammatory diseases?

Our response: The reviewer asks here important questions that should be followed up. However, they are beyond the scope of this manuscript and would require a dedicated research effort spanning years. Most importantly, we need to isolate TM7 strains from the gut microbiome and directly explore their colitogenic potential. We feel exploring the microbiota from other facilities is not going to provide a definitive answer. We hope that our manuscript will incite more labs to explore the connection between TM7 and colitis in the mouse model and human. However, there is already published evidence that highlights a link between TM7 and colitis in human and we describe this in the Discussion section, writing:

*“A potential link between TM7 strains and Crohn’s disease has been previously described [58]. TM7 levels are regulated by the inflammasome [59], have been linked to inflammatory activity of the microbiome in aged mice [60] and changes in the mucus layer barrier function of the distal colon [61].”*

**Comment 7.**      Overall, this is a very descriptive study and the statements related to Smarcad1 and microbiota-induced inflammation are overstated given the lack of convincing data to support the conclusions drawn by the authors.
**Our response:**

We have now changed the title of the paper, also following the advice of reviewer 2:

Smarcad1 mediates microbiota-induced inflammation AND coordinates gene expression in the intestinal epithelium

**Reviewer #2:** The authors have adequately addressed most comments.

**Comment 1.** The title should be modified to reflect the more accurate interpretation that they now use in the abstract/text (Rev 2, Comment 1). The original title indicates causation, but a title similar to below would be in line with the data presented.

Smarcad1 mediates microbiota-induced inflammation AND coordinates gene expression in the intestinal epithelium

Our response: We have changed the title now as advised.

**Comment 2**. The authors include colon analyses for H3K9me3, and gene expression (new Fig. 4). This seems necessary given that DSS is a model of colitis. However, they note very little correlation between H3K9me3 and DEG. How do the authors interpret this finding and how do these DEGs relate to those genes observed during microbiota-enriched colitis that they describe in Fig 8?

**Our response:**

Please, see also our response to comments 2,3 and 4 of reviewer 1. While the number of genes that show significant changes in H3K9me3 in the colon epithelium AND changes in gene expression is low, albeit statistically significant (Figure 4c), we do see a general increase in H3K9me3 in downregulated genes in the colon epithelium (something we did not detect in the small intestinal epithelium).

**Third round of review**

**Reviewer 1**

The authors have adequately addressed the criticisms raised and the manuscript is significantly improved.
